# Chemical composition’s effect on *Solanum nigrum* Linn.’s antioxidant capacity and erythrocyte protection: Bioactive components and molecular docking analysis

**DOI:** 10.1515/biol-2022-0944

**Published:** 2024-08-30

**Authors:** Abdelatif Aouadi, Djamila Hamada Saoud, Abdelkrim Rebiai, Mona H. Ibrahim, Mohammed Messaoudi, Khaoula Alia, Halima Zidane, Ayomide Victor Atoki, Fatma Mohamed Abd El-Mordy

**Affiliations:** Process Engineering Laboratory, Applied Sciences Faculty, Kasdi Merbah University, Ouargla, 30000, Algeria; Laboratory of Applied Chemistry and Environment, Faculty of Exact Sciences, University of Hamma Lakhdar El-Oued, B.P.789, 39000, El-Oued, Algeria; Department of Pharmaceutical Medicinal Chemistry and Drug Design, Faculty of Pharmacy (Girls), Al-Azhar University, Cairo, 11754, Egypt; Nuclear Research Centre of Birine, P.O. Box 180, Ain Oussera, Djelfa, 17200, Algeria; Department of Process Engineering and Petrochemical, Faculty of Technology, University of El Oued, 39000, El-Oued, Algeria; Department of Biochemistry, Kampala International University, Ishaka, Uganda; Department of Pharmacognosy and Medicinal Plants, Faculty of Pharmacy (Girls), Al-Azhar University, Cairo, 11754, Egypt

**Keywords:** *Solanum nigrum*, phenolic, flavonoids, antioxidant activity, DPPH assay, FRAP assay

## Abstract

Oxidative stress has been widely believed to be the mechanism responsible for developing diseases such as arthritis, asthma, dementia, and aging. *Solanum nigrum* Linn. is a common edible medicinal herb that belongs to the family Solanaceae which has more than 180 chemical components that have so far been discovered. The main bioactive components of these are steroidal saponins, alkaloids, phenols, and polysaccharides. This article presents comparative phytochemical profiling including total phenolic, total flavonoid, alkaloid, proanthocyanidins, tannin, and vitamin C contents of three Algerian *S. nigrum* samples collected from three different locations in the Algerian desert. Additionally, the potential antioxidant activity of the three samples was assessed by 2,2-diphenyl-1-picrylhydrazyl, ferric reducing antioxidant power, and oxidative hemolysis inhibition assay. Moreover, the correlation between the major phenolic phytoconstituents previously reported and isolated from the plant and antioxidant activity has also been done by *in silico* molecular docking. Ten bioactive compounds were docked with selected proteins, arachidonate-5-lipoxygenase (PDB: 6n2w) and cytochrome *c* peroxidase (PDB: 2x08), to check their affinity with binding sites of these proteins for the possible mechanism of action. The docking scores suggest that *S. nigrum*’s quercetin and kaempferol may play a significant role in its antioxidant action.

## Graphical abstract

**Figure:**
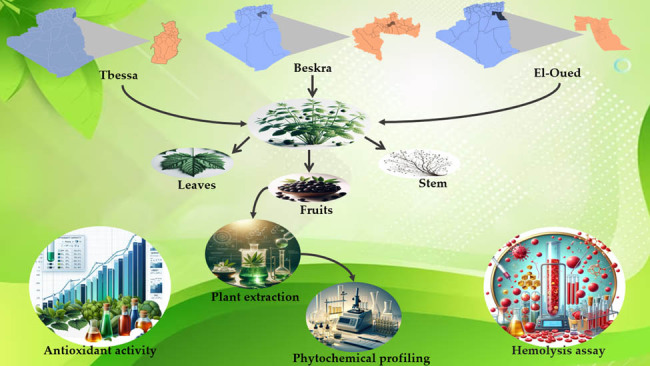

## 1 Introduction

Reactive oxygen species (ROS) and reactive nitrogen species are generated by natural biological processes as well as by external factors such as radiation, pollution, cigarette smoke, and medication [1]. These two classes of reactive species break down carbohydrates, lipids, proteins, and nucleic acids [2]. Although these reactive species play advantageous physiological roles at low to moderate concentrations, they are harmful and lead to oxidative stress at higher quantities [3]. Oxidative stress is worsened by high levels of free radical production and insufficient endogenous antioxidant defense in the human body. This leads to cellular damage, ageing, diabetes, cancer, neurological illness, and cardiovascular disease [4]. Therefore, exogenous dietary antioxidants can attenuate or stop this oxidative stress-induced degenerative illness. Numerous bioactive substances that can be derived from plants or found in functional meals are being studied for their potential to treat serious, complex disorders including cancer [5]. There are two types of antioxidants: synthetic antioxidants and natural antioxidants. Propyl gallate, tributyl hydroquinone, butylated hydroxyl toluene, and butylated hydroxyl anisole are among the first set of chemicals that harm humans’ livers and cause cancer [6]. The recent focus of the food and pharmaceutical industries has been to replace these synthetic antioxidants with natural antioxidants that have beneficial medical, nutritional, and safe properties. The body is protected from free radicals by natural antioxidants that have been taken from plant sources that are high in phenolic, flavonoids, vitamin C, carotenoids, tannins, and proanthocyanins [7]. These plant sources include edible wild plants, which may contain undiscovered nutritional and bioactive components that can reduce oxidative stress [8]. Natural chemicals derived from plants are viewed as superior substitutes to manufactured medications since they are cheaper, less harmful, and less expensive [9]. The existence and quantity of phytochemicals like alkaloids, tannins, flavonoids, and phenolic compounds that these plants synthesize are what make them helpful as therapeutic species [10].

*Solanum nigrum* (L.) is a herbaceous plant which is widespread around the world and also a great source of phytochemicals [11]. Black nightshade is the English name for *S. nigrum* L., while Makoi, Kachcipandu, Munatakali, Piludi, and Kamuni are the Hindi, Gujrati, Telugu, Tamil, and Marathi names for the plant [12]. *S. nigrum* has naturalized in Africa despite being native to Eurasia and imported to the Americas, Australasia, and South Africa. It is now a part of the flora known for several health advantages [13]. This plant has a long history of use in the traditional treatment and prevention of numerous illnesses. It has long been connected to analgesic properties and the treatment of tracheitis, cancer, liver damage, mastitis, and aerodontalgia [14]. Despite the plant’s numerous health benefits, it is only accessible in the wild, and its population is rapidly falling due to human activity, including habitat modification, chemical eradication, displacement for the cultivation of farmer’s chosen field crops, and others [14].

Many steroidal glycosides, steroidal alkaloids, steroidal oligoglycosides, such as solamargine, solasonine, solavilline, and solanine, as well as steroidal saponins and glycoprotein, as well as a number of polyphenolic compounds, including gallic acid, protocatechuic acid, catechin, caffeic acid, epicatechin, rutin, and naringenin, have been previously reported in *S. nigrum* L., which possess strong antioxidant, anticancer activity, and HIV reverse transcriptase inhibitors [15].

Many studies have reported that there is a direct relationship between biological activities and therapeutic properties of plants [16], and it is also understood that the biological attributes of the plant could be linked to the available resources in the soil for plant uptake. However, no report has been made on the influence of soil texture types on the phytochemical and antioxidant properties of *S. nigrum.* Thus, this study therefore hypothesized that modulation of soil texture does influence the phytochemical constituents and antioxidant properties of *S. nigrum* cultivated in glasshouses.

The aim of this study was to investigate the total phenolic content (TPC), total flavonoid content (TFC), and vitamin C content of three Algerian *S. nigrum* samples collected from three different places. Furthermore, the study determines the alkaloid, proanthocyanidins, and tannin contents in the three different plant samples. This phytochemical investigation was followed by assessing its potential antioxidant activity by 2,2-diphenyl-1-picrylhydrazyl (DPPH), ferric reducing antioxidant power (FRAP), and oxidative hemolysis inhibition assay. Moreover, the correlation between phytoconstituents reported in the plant and antioxidant activity has been done by *in silico* molecular docking.

## 2 Materials and methods

### 2.1 Plant material

*S. nigrum* L. was collected in the summer of 2022 from eastern Algeria, from three sites listed in Table 1. *S. nigrum* L. was identified by Pr. Chehma A. (Ouargla University) and a voucher specimen (SN-12-22) was placed in the Laboratory of Process Engineering, Faculty of Applied Sciences, Kasdi Merbah University, Ouargla, Algeria. Then, the plant samples were dried, crushed, and kept away from moisture and light.

**Table 1:** Sites of plant collection

Collection area	Plant organ	Abbreviation
Tebessa	Fruits	TF
	Leaves	TL
	Stem	TS
Biskra	Fruits	BF
	Leaves	BL
	Stem	BS
El-Oued	Fruits	EF
	Leaves	EL
	Stem	ES

### 2.2 Plant extraction

The plants were completely dried using a freeze dryer (Alpha 1-4 LD plus, Christ, Osterode, Germany). The dried powdered plant samples were extracted using a modified version of the method of Aouadi et al. [17], with some modifications. About 0.5 g of each sample was soaked for 24 h with 50 mL of methanol (99.8% absolute methanol) in a capped bottle and shaken in an orbital shaker at 180 rpm at room temperature. The agitated sample was centrifuged at 10,000 rpm for 10 min and the supernatant was recovered.

### 2.3 Phytochemical profiling

#### 2.3.1 Determination of TPC

Using a modified version of Aouadi et al.’s 2003 approach, the total phenols in the methanol extract were assessed spectrophotometrically using Folin–Ciocalteu reagent [16]. About 5 mL of Folin–Ciocalteu reagent that had been diluted 1:9 with water was added to the extracts. About 4 mL of sodium carbonate (75 g/L) was added to this mixture, vortexed for 10 s, and then left to stand for 30 min at 40°C to develop color. The UV–Vis 3000 PC spectrophotometer was used to measure the absorbance at 765 nm. The extracts’ TPC was calculated as mg of tannic acid equivalents (TAE) per gram of dry sample weight (mg/g). The experiment was conducted in triplicate and the results were expressed as mean standard deviation (SD) values. The TPC was calculated as TAE by the following equation:

\[T=\frac{C\times V}{M},]\]

where *T* is the TPC in mg/g of the extracts as TAE, *C* is the concentration of tannic acid established from the calibration curve in mg/mL, *V* is the volume of the extract solution in mL, and *M* is the weight of the extract in g.

#### 2.3.2 Determination of TFC

The TFCs of all the extracts were quantified following the protocol outlined by Hailong et al. [18]. Initially, 700 μL of all the plant extracts were taken in different test tubes. To each extract, 2 mL of distilled water was added. Then, 150 μL of NaNO_2_ was added to each test tube followed by incubation at room temperature for 6 min. After incubation, 150 μL of AlCl_3_ (10%) was added to each test tube. The test tubes were incubated for 6 min at room temperature. Then, 2 mL of 4% NaOH was added to all the test tubes which were made up to 5 mL using distilled water. The contents in all the test tubes were vortexed well and they were allowed to stand for 15 min at room temperature. The presence of flavonoids was read with a spectrophotometer at 510 nm. The level of flavonoids was calculated as milligram catechin equivalent per gram (mg CE/g).

#### 2.3.3 Determination of alkaloid content

Crude samples of *S. nigrum* extracts were subjected to quantitative analysis using a modified version of Harborne’s (1980) methodology [19]. Five grams of the *S. nigrum* sample was mixed with 20 mL of 10% acetic acid (Ac) prepared in ethanol, then capped and allowed to sit for 4 hours. The combination was filtered, and the filtrate was then concentrated in a water bath to a quarter of its original volume. Drop by drop, concentrated ammonium hydroxide was added to the extract until the precipitation was complete. The entire solution was left to settle and washed with diluted ammonium hydroxide before being re-filtered. The weight of the dried residue was determined, and the percentage composition was calculated using the equation:

\[{\mathrm{Alkaloids}}( \% )=\left(\frac{{\mathrm{final\; weight\; of\; the\; residue}}}{{\mathrm{initial\; weight\; of\; the\; sample}}}\right)\times 10.]\]

#### 2.3.4 Determination of proanthocyanidin content

The total proanthocyanidin content of *S. nigrum* was determined using the procedure reported by Zhang et al. [20]. A total of 3.0 mL of (4% v/v) vanillin methanol solution, 1.5 mL of hydrochloric acid, and 0.5 mL of the extract at 0.1 mg/mL were added together and vortexed. The combination was left at room temperature for 15 min, after which the absorbance at 500 nm was determined. Total proanthocyanidin content was calculated as catechin equivalents (CEs) (mg/g) using the equation from the calibration curve: *Y* = 0.5825*x*, *R* ^2^ = 0.9277, where *x* is the concentration of the CE and *Y* is the absorbance.

#### 2.3.5 Determination of tannin contents

Tannin content was determined in plants from different soil types using the method of Irina et al. [21] with some modifications. A mass of 0.20 g of *S. nigrum* methanol extract was added to 20 mL of 50% methanol, vortexed vigorously, and incubated in the water bath for 1 h at 80°C and filtered. The filtrate was poured in 20 mL of distilled water. About 2.5 mL of *Folin-Denis* reagent was then mixed with 10 mL of 17% aqueous NaCO_3_, mixed with the filtrate, and made up to 100 mL with distilled water. The mixture was allowed to react for 20 min. At the end of the reaction, there was a bluish-green color that indicated the presence of tannins. Absorption of the tannic standard solution and the samples were then measured at 706 nm. Results were expressed in mg/g of TAE to the calibration curve: *Y* = 0.0593*x*0.0485, *R* ^2^ = 0.9826, where *x* is the absorbance and *Y* is the TAE.

### 2.4 Determination of vitamin C content

The vitamin C content was determined according to the official methods of analysis [22], 2,6-dichloroindophenol titration method 967.21. Briefly, a 0.1 g powder sample was extracted with 40 mL of 15 g of metaphosphoric acid (HPO_3_) and mixed with 40 mL of Ac in 500 mL of deionized H_2_O. The extracted sample was filtered using the Whatman number one filter paper. The filtrated sample was titrated using an indophenol solution made by dissolving 50 mg of 2,6-dichloroindophenol sodium salt and 42 mg of NaHCO_3_ in 200 mL of deionized water. The mixture was filtered through Whatman number one filter paper into the amber bottle and stored in a refrigerator until use.

The standard solution of vitamin C was prepared by transferring 50 mg of vitamin C into 50 mL flask and diluted to volume using freshly prepared HPO_3_(Ac). The vitamin C content was calculated according to the following equation:

\[{\mathrm{vitamin\; C}}({\mathrm{mg}}/{\mathrm{g}})=\frac{(A\left-B)\times C\times 40}{10\times S},]\]

where *A* = volume in mL of the 2,6-dichloroindophenol sodium salt solution used for the sample; *B* = volume in mL of the 2,6-dichloroindophenol sodium salt solution used for the blank; *C* = mass in mg of l ascorbic acid equivalent to 1.0 mL of standard indophenol solution; *S* = weight of a sample taken (g); 40 = volume of extract; and 10 = volume of extract used for the determination.

### 2.5 Antioxidant activity

#### 2.5.1 Determination of antioxidant activity by DPPH assay

The determination of DPPH stable radical scavenging activities of the extracts and standards was evaluated based on the method described by Aouadi et al. [23]. Extracts (1 mL) of different concentrations (0.2–0.56 mg/mL) made by reconstituting in respective solvents were added to DPPH solution (5 mL, 0.1 mM) in methanol and vortexed. After 20 min of reaction at 25°C, the absorbance was measured at 517 nm against a blank (methanol) in a UV–Vis spectrophotometer (Shimadzu UV-2401PC). Methanolic DPPH solution (5 mL) without antioxidants was used as a control. The DPPH scavenging activity of the extract was expressed as IC_50_ (inhibitory concentration), that is, the concentration of the extract at which DPPH radicals were quenched by 50%. Ascorbic acid was used as the standard antioxidant. The percentage quenching of DPPH was calculated as follows:

\[{\mathrm{DPPH\; inhibiting\; capacity}}( \% )=\left(\frac{{{\mathrm{Abs}}}_{{\mathrm{control}}}-{{\mathrm{Abs}}}_{{\mathrm{sample}}}}{{{\mathrm{Abs}}}_{{\mathrm{control}}}}\right)\hspace{13.5em}\times 100.]\]

#### 2.5.2 Determination of antioxidant activity by FRAP

The FRAP analysis was conducted based on a modified method of Carducci et al. [24]. Briefly, the FRAP reagent was made by mixing acetate buffer (pH 3.6, 300 mL), 10 mMol/L 2,4,6-tri-2-pyridyl-s-triazine, and 20 mmol/L FeCl_3_ at a ratio of 10:1:1 (v/v/v). Then, 150 μL of the methanol extract solution of *S. nigrum* was mixed with 4.5 mL of the FRAP reagent, and the mixture was incubated in a water bath at 37°C for 10 min. The absorbance of the reacted mixture was measured by the UV–Vis spectrophotometer (Shimadzu UV-2401PC) at 593 nm, and the FRAP of each sample was calculated using a calibration curve with Fe2 (Sigma-Aldrich Co, USA) as the standard.

#### 2.5.3 Oxidative hemolysis inhibition assay

This test is used to determine the ability of the plant extracts to protect the erythrocyte blood cells from damage or disruption of the cell membrane after exposing them to oxidative stress and free radicals by measuring the percentage of dissolved erythrocytes [25]. According to Chouikh et al. [26], a volume of 40 µL of human erythrocytes was mixed with 2 mL of different concentrations (0.25–1 mg/mL) of *S. nigrum* extract and conserved for 5 min at 37°C. Then, we added 40 µL of H_2_O_2_ (30 × 10^−3^ M), 40 µL of FeCl_3_ (80 × 10^−3^ M), and 40 µL of ascorbic acid solution (50 × 10^−3^ M), respectively. After 1 h of incubation at 37°C, the mixture was centrifuged with 700 rot/min for 10 min. The absorbance of the supernatant was read at *λ* = 540 nm. The percentage of hemolysis was determined using the following formula:

\[{\mathrm{Hemolysis\; inhibition}} \% =\left(\frac{{{\mathrm{Abs}}}_{{\mathrm{control}}}}{{{\mathrm{Abs}}}_{{\mathrm{sample}}}}\right)\times 100.]\]

### 2.6 Docking study

The docking analysis was performed with the Autodock vina 4.2 software. The derivatives were subsequently submitted to energy minimization using the default MMFF94x force. The three-dimensional conformations of these compounds were acquired through the application of a conformer search methodology. The lipoxygenase (LOX) and peroxidase enzymes were obtained from the Protein Data Bank (PDB) with the corresponding identification codes 6n2w and 2x08, respectively. Water molecules were eliminated from the software, and any hydrogen atoms that were absent were replaced to guarantee the protein structure maintained accurate ionization states. We utilized the Biovia discovery-studio 2021 visualizer to examine the representation of protein–ligand interactions in the active site of the complex. In conclusion, the H bonds, hydrophobic, and Pi–Pi interactions are regarded as the most significant ones [27,28].

### 2.7 Statistical analysis

The data presented represent the average of three replicates with SD.

A multiway analysis of variance was applied to the results and mean comparisons were performed using Tukey’s multiple range test with SPSS version 20.0 (Statistical Package for the Social Sciences, Inc., Chicago, IL, USA). Significance was considered at *p* < 0.05. To determine the relationship between the chemical content of plants and the relationship between the chemical content of plants and antioxidants or other biological activities, the linear correlation coefficient was calculated. For exploratory data analysis, the results were processed through one of the multivariate analysis techniques, principal component analysis (PCA). PCA was performed using XLSTAT (version 2020.1, Addinsoft, Pearson edition, Waltman, MA, USA) to enhance discrimination between the studied parameters.

A multiway analysis of variance (ANOVA) was utilized to evaluate the effects of multiple factors and their interactions on the outcome variables simultaneously. This statistical method allows researchers to determine the individual impact of each factor on the dependent variable, while also identifying how different factors interact with one another. By accounting for multiple sources of variation, multiway ANOVA increases the statistical power, enhancing the ability to detect significant differences. Additionally, this approach helps control for confounding variables, thereby reducing potential bias and leading to more accurate and reliable conclusions.

## 3 Results and discussion

### 3.1 Quantitative estimation of TPC

**Figure 1:**
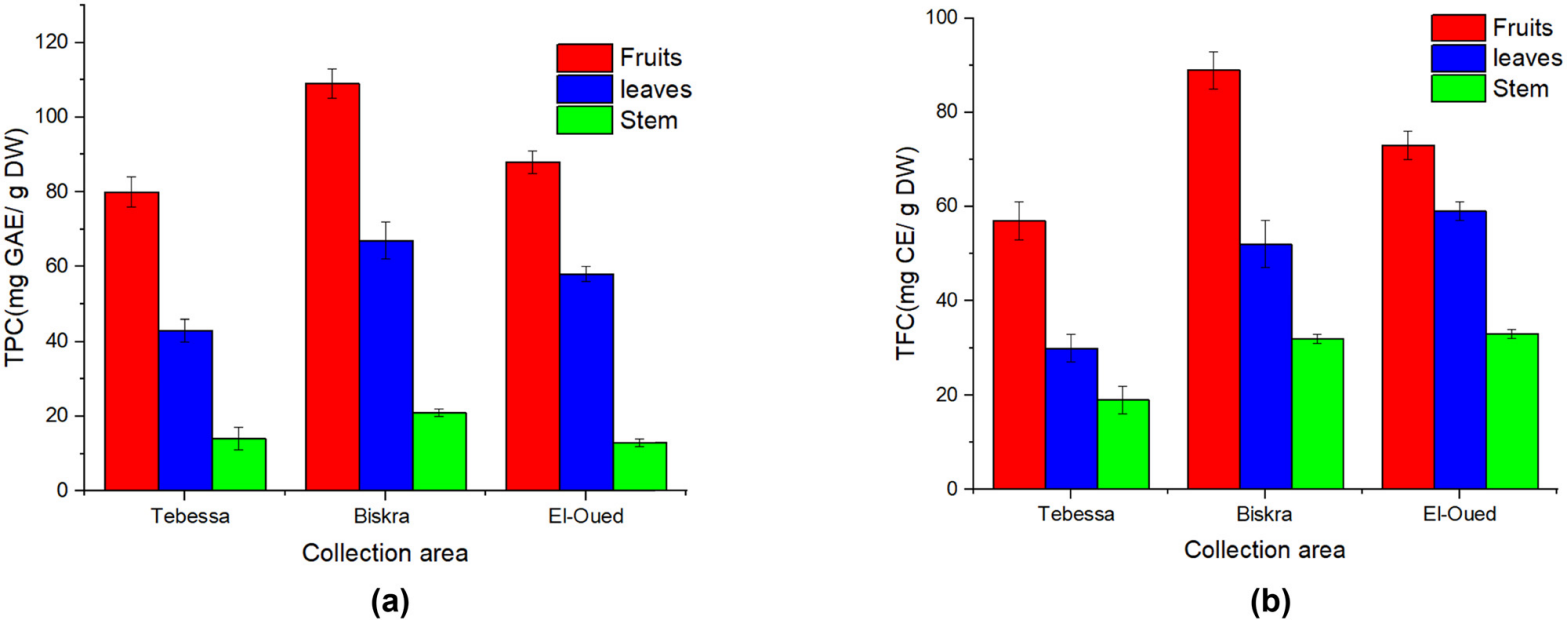
(a) TPC and (b) TFC of *S. nigrum* extracts.

Phenolic compounds are known to be potent chain-breaking antioxidants that serve as free radical terminators, which may directly contribute to antioxidative action. These chemicals are very significant plant ingredients, and their radical scavenging ability is related to the presence of hydroxyl groups in them. Extracts with significant antioxidant activity have a high phenolic content in general. Polyphenols are the most abundant plant chemicals with antioxidant activity due to their redox characteristics; they are vital in absorbing and neutralizing free radicals, quenching singlet and triplet O_2_, and degrading peroxides. Plants in phenols have antioxidant, anti-mutagenic, and anti-cancer effects [29]. The TPC of three different samples of *S. nigrum* L. fruits, leaves, and stem was determined by the *Folin*–*Ciocalteu* method [30]. The TPC of extracts ranged from 11.3 to 109.2 mg of gallic acid equivalent (GAE)/g on a dry weight (dw) basis (Table 2). TPC levels were highest in the Biskra fruit (BF) extract of *S. nigrum*, and lowest in El-Oued leaf (EL) extract of *S. nigrum* (Figure 1a).

**Table 2:** Total phenolic, total flavonoid, and phytochemical profile of the wild *Solanum nigrum* L. collected from three different locations in Algerian desert

*S. nigrum* extract	TPC (mg GAE/g)	TFC (mg CE/g)	Tannin content (mg TE/g)	Proanthocyanidin content (mg CE/g)	Vitamin C (mg/100 g)	Alkaloids (%)
TF	79.87 ± 0.10^c^	58.23 ± 0.12^cd^	93.37 ± 0.14^b^	861.33 ± 0.10^c^	32.27 ± 0.12^c^	37.67 ± 0.12^d^
TL	17.6 ± 0.09^h^	29.07 ± 0.12^f^	56.30 ± 0.05^e^	557.77 ± 0.17^f^	18.37 ± 0.12^fg^	24.80 ± 0.14^h^
TS	53.43 ± 0.12^f^	19.77 ± 0.12^h^	40.07 ± 0.17^g^	220.30 ± 0.09^i^	15.33 ± 0.12^h^	22.23 ± 0.11^i^
BF	109.17 ± 0.17^a^	88.33 ± 0.12^a^	97.17 ± 0.12^a^	889.67 ± 0.07^a^	47.07 ± 0.12^a^	50.97 ± 0.19^ab^
BL	40.1 ± 0.14^g^	51.23 ± 0.07^e^	74.47 ± 0.10^c^	697.57 ± 0.07^de^	34.77 ± 0.12^b^	35.20 ± 0.19^e^
BS	76.30 ± 0.09^d^	22.47 ± 0.12^g^	29.73 ± 0.07^h^	331.67 ± 0.07^h^	27.40 ± 0.14^d^	26.70 ± 0.09^g^
EF	83.47 ± 0.17^b^	74.30 ± 0.08^b^	92.83 ± 0.17^b^	881.43 ± 0.14^ab^	34.17 ± 0.17^b^	46.33 ± 0.15^c^
EL	11.33 ± 0.12^i^	59.67 ± 0.12^c^	64.43 ± 0.14^d^	708.37 ± 0.14^d^	21.63 ± 0.10^e^	51.03 ± 0.14^a^
ES	63.67 ± 0.15^e^	19.63 ± 0.10^h^	42.77 ± 0.14^f^	490.30 ± 0.12^g^	19.90 ± 0.17^f^	33.47 ± 0.19^f^

Values with different superscript letters are statistically different.

### 3.2 Quantitative estimation of TFC

Flavonoids are likely the most important natural phenolics due to their wide range of chemical and biological activity, including antioxidant and free radical scavenging characteristics. Flavonoids have been described as antioxidants, scavengers of a wide range of ROS, and regulators of lipid peroxidation, as well as potential therapeutic agents against a wide range of disorders [29]. The TFC of the nine *S. nigrum* extracts was expressed as mg CE/g dw extract. The TFC of extracts ranged from 19.7 to 88.3 mg CE/g (Table 2). BF and El-Oued fruit (EF) extracts of *S. nigrum* contained the most TFC, whereas El-Oued stem (ES) extract and Tebessa stem (TS) extract contained the least (Figure 1).

### 3.3 Quantitative estimation of proanthocyanidin content

**Figure 2:**
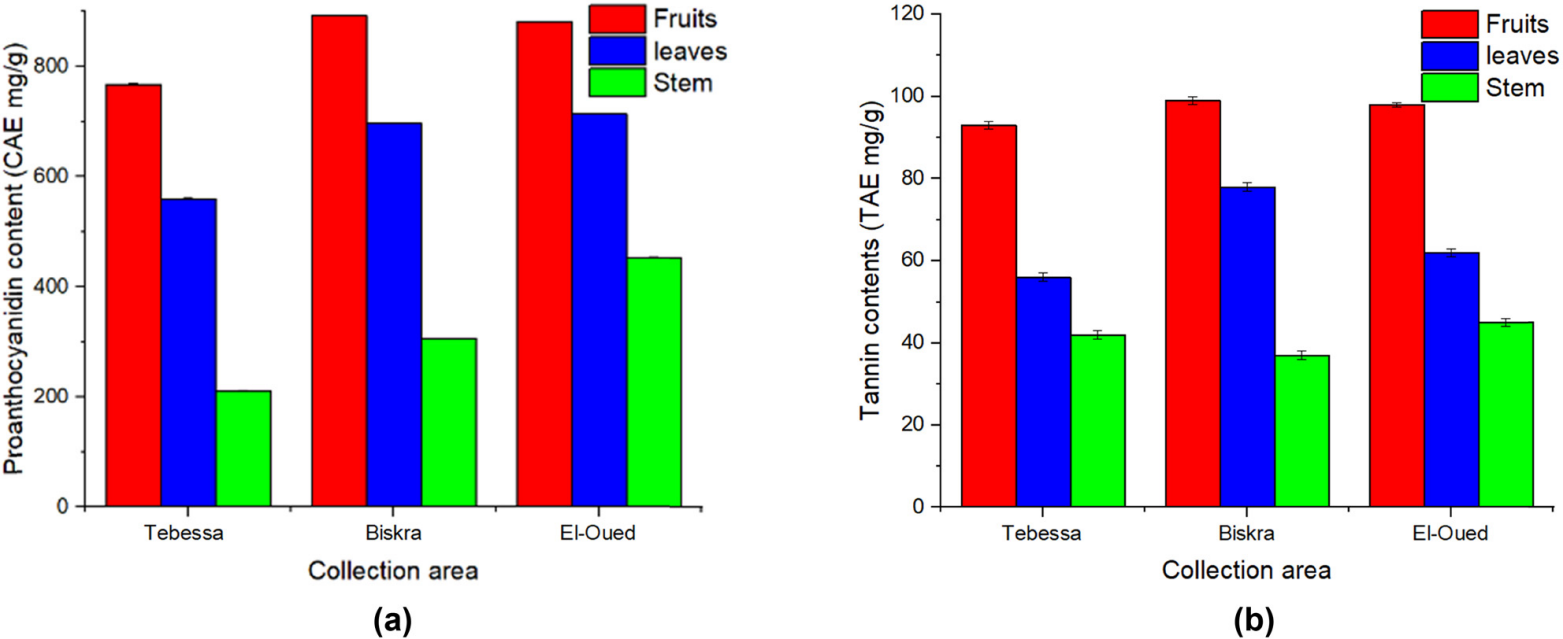
(a) Proanthocyanidin content and (b) tannin content in *S. nigrum* extracts.

Proanthocyanidins are flavan-3-ol-based tannins that are condensed or nonhydrolyzable. These chemicals are extensively spread throughout nature, including vegetables, fruits, and plants. Proanthocyanidins have been shown to have potent free-radical scavenging and antioxidative properties. Many significant biological actions, such as anticancer, antiviral, and anti-inflammatory, have been reported [31]. Total proanthocyanidin content was calculated as CE (mg/g) as listed in Table 2. The study results for proanthocyanidin concentration of *S. nigrum* L. extracts ranged from 220.3 to 889.7 mg CE/g. Fruits of *S. nigrum* L. had the highest proanthocyanidin concentration, followed by *S. nigrum* L. leaves, while *S. nigrum* L. stem had the lowest as shown in Figure 2a.

### 3.4 Quantitative estimation of tannin content

Tannins are classified as polyphenols due to the abundance of phenolic rings in their structures. These natural compounds can be found in plant tissues such as bark, fruit, and wood and can be extracted from these sources using water [32]. The tannin content of *S. nigrum* L. was expressed in mg/g of TAE. From Table 2, tannin content was highest in the fruits of *S. nigrum* L. rather than leaves and stem. Fruits from Biskra region (BF) were the richest followed by fruits from Tebessa (TF) and El-Oued (EF) regions. While the stem of *S. nigrum* L. from Tebessa (TS) contains the lowest concentration of tannins (Figure 2b).

### 3.5 Quantitative estimation of alkaloid content

**Figure 3:**
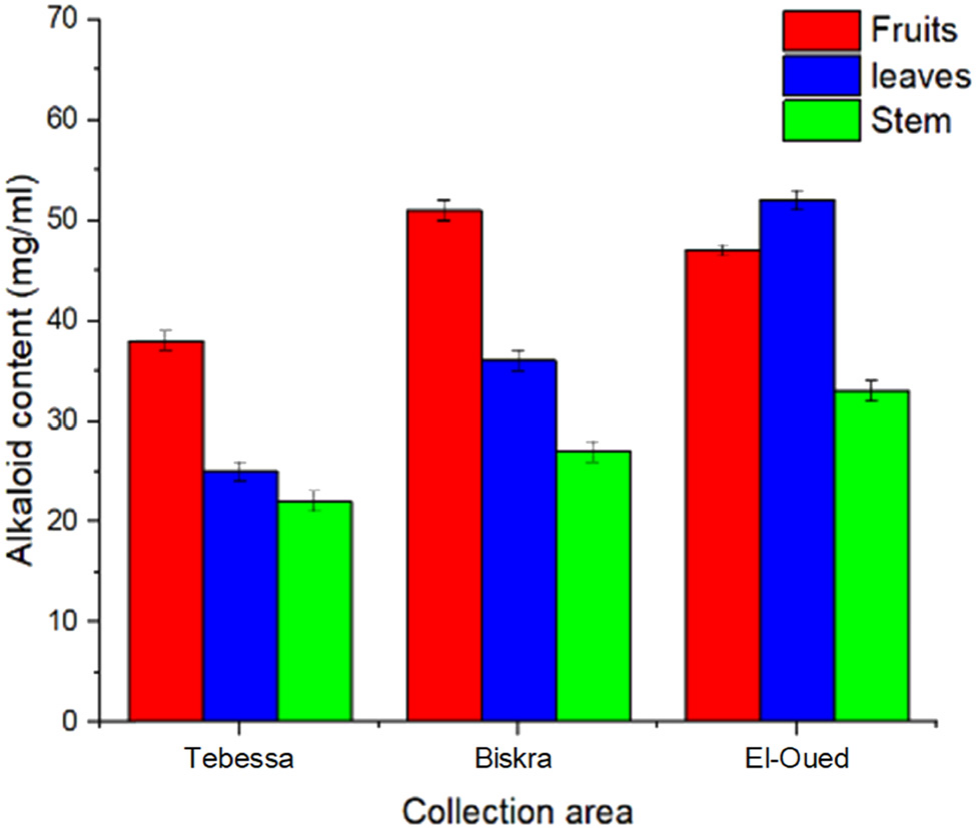
Alkaloids content of *S. nigrum* extract.

Alkaloids have always piqued the curiosity of scientists due to their good or bad impacts on humans. Surprisingly, many alkaloids can act as both anti-oxidants and pro-oxidants depending on the circumstances [33]. The results showed that *S. nigrum* leaf from El-Oued region (EL) had the maximum alkaloid concentration (51.1%), followed by *S. nigrum* fruits from Biskra region (BF) (50.9%) and *S. nigrum* fruits from El-Oued region (EF) (46.2%). Stem of *S. nigrum* collected from Tebessa region (TS) has the lowest alkaloid concentration (22.1%) (Figure 3).

### 3.6 Quantitative estimation of vitamin C content

**Figure 4:**
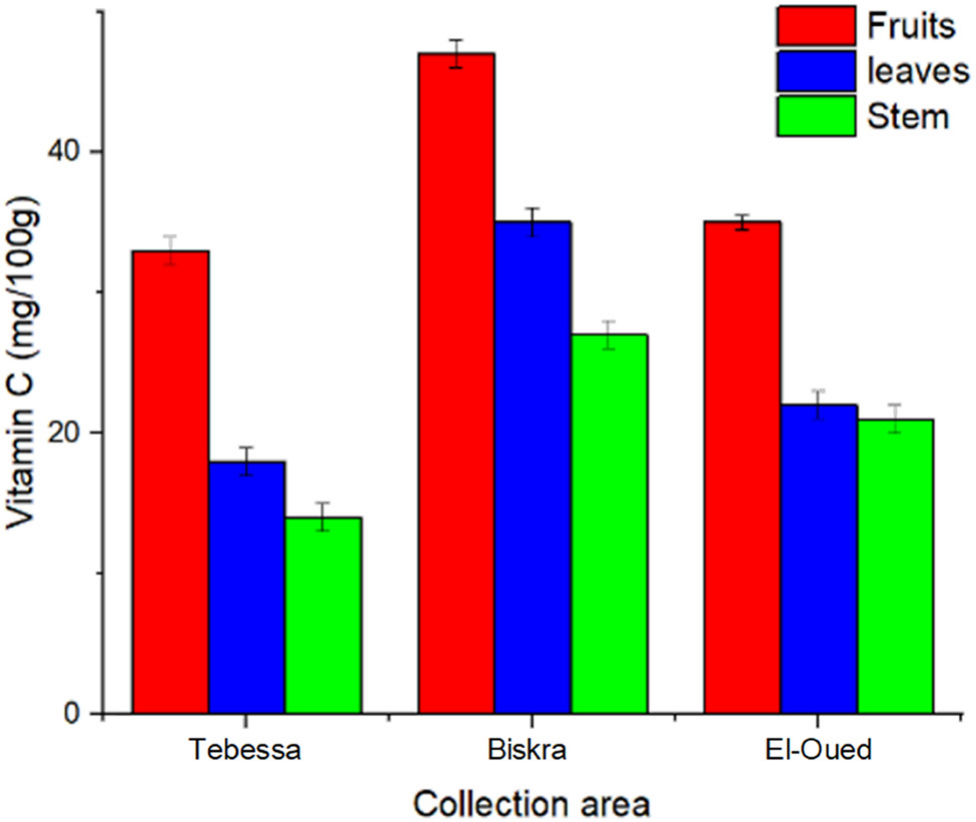
Vitamin C content of *S. nigrum* extracts.

As demonstrated in Table 2, the vitamin C content of three different samples of *S. nigrum* L. collected from Tebessa, Biskra, and El-Oued differed somewhat. The vitamin C content of the nine extracts ranged from 15.3 mg/100 g (dw) in the stem extract of *S. nigrum* collected from Tebessa (TS) to 47.1 mg/100 g (dw) in the fruit extract of *S. nigrum* collected from BF. The maximum vitamin C concentration was identified in the fruits of *S. nigrum* collected from BF, followed by *S. nigrum* fruits collected from El-Oued (EF), while the lowest quantity was found in the stems collected from TS (Figure 4).

### 3.7 PCA

**Figure 5:**
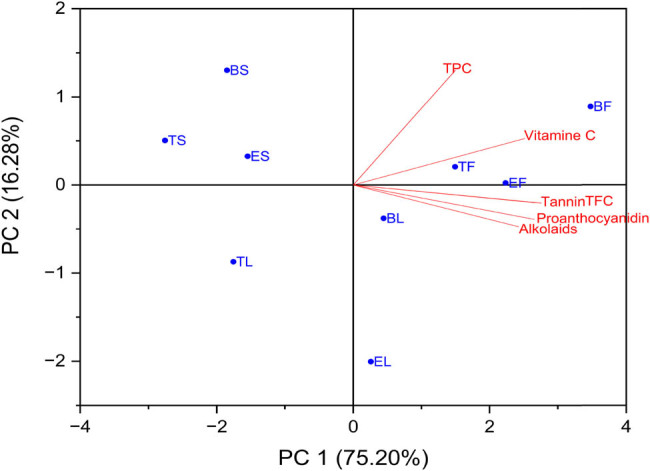
Biplot PCA of TPC, TFC, tannin, proanthocyanidins, vitamin C, and alkaloids for the nine plant extracts. The percentages of PC1 and PC2 represent the largest and second largest variance in the data on the *X*-axis and *Y*-axis, respectively.

Using PCA, we were able to acquire a more in-depth understanding of the link between the overall amount of various chemical components that were computed for each of the nine extracts. Through the use of this study, the purpose was to shed light on the relationships that exist between the nine plant extracts’ six primary components, which are as follows: TPC, TFC, tannin, proanthocyanidins, vitamin C, and alkaloids. Figure 5 and Table 3 illustrate this point.

**Table 3:** Correlation matrix between chemical content of plant extracts

	TPC	TFC	Tannin	Proanthocyanidins	Vitamin C	Alkaloids
TPC	1	0.40335	0.3869	0.29468	0.68767	0.24912
TFC	0.40335	1	0.92158	0.90794	0.82593	0.88579
Tannin	0.3869	0.92158	1	0.95028	0.76529	0.72839
Proanthocyanidins	0.29468	0.90794	0.95028	1	0.73841	0.81997
Vitamin C	0.68767	0.82593	0.76529	0.73841	1	0.63174
Alkaloids	0.24912	0.88579	0.72839	0.81997	0.63174	1

With a correlation value of 0.68767, the total polyphenol content was shown to have a substantial positive link with vitamin C. On the other hand, the correlations with the remaining chemicals ranged from weak to moderate. The flavonoid content had a substantial and positive association with all of the components, with the correlation coefficients being 0.92158, 0.3869, and 0.90794 for TPC, tannin, proanthocyanidins, vitamin C, and alkaloids, respectively (as shown in Table 3). It was also found that the flavonoid content was favorably connected with all of the components.

Figure 5 demonstrates that a very significant positive correlation ratio was found between proanthocyanidin and tannin and TFC. This connection was documented and demonstrated by the structural inputs. The results of the study are presented in Table 4, which shows that PC1 had a significant positive correlation with TFC (*r* = 0.45874), but PC2 only had a high and positive correlation with TPC (*r* = 0.83556) and vitamin C (*r* = 0.35783).

**Table 4:** Correlation between variables (chemical content) and factors for plant extracts

	Coefficients of PC1	Coefficients of PC2
TPC	0.24891	0.83556
TFC	0.45874	−0.13163
Tannin	0.44199	−0.12841
Proanthocyanidin	0.4412	−0.2493
Vitamin C	0.41827	0.33783
Alkaloids	0.4033	−0.30288

The results of Tukey’s test (Table 2) confirm that there are large variations between the harvest area and the plant part with regard to their effectiveness in extracting the active substances studied, as it is clear that the BF sample is the best sample. Therefore, the Tebessa region is the best harvesting area, and the leaf part is the best part of the plant.

### 3.8 Antioxidant activity

#### 3.8.1 Determination of antioxidant activity by DPPH assay

**Figure 6:**
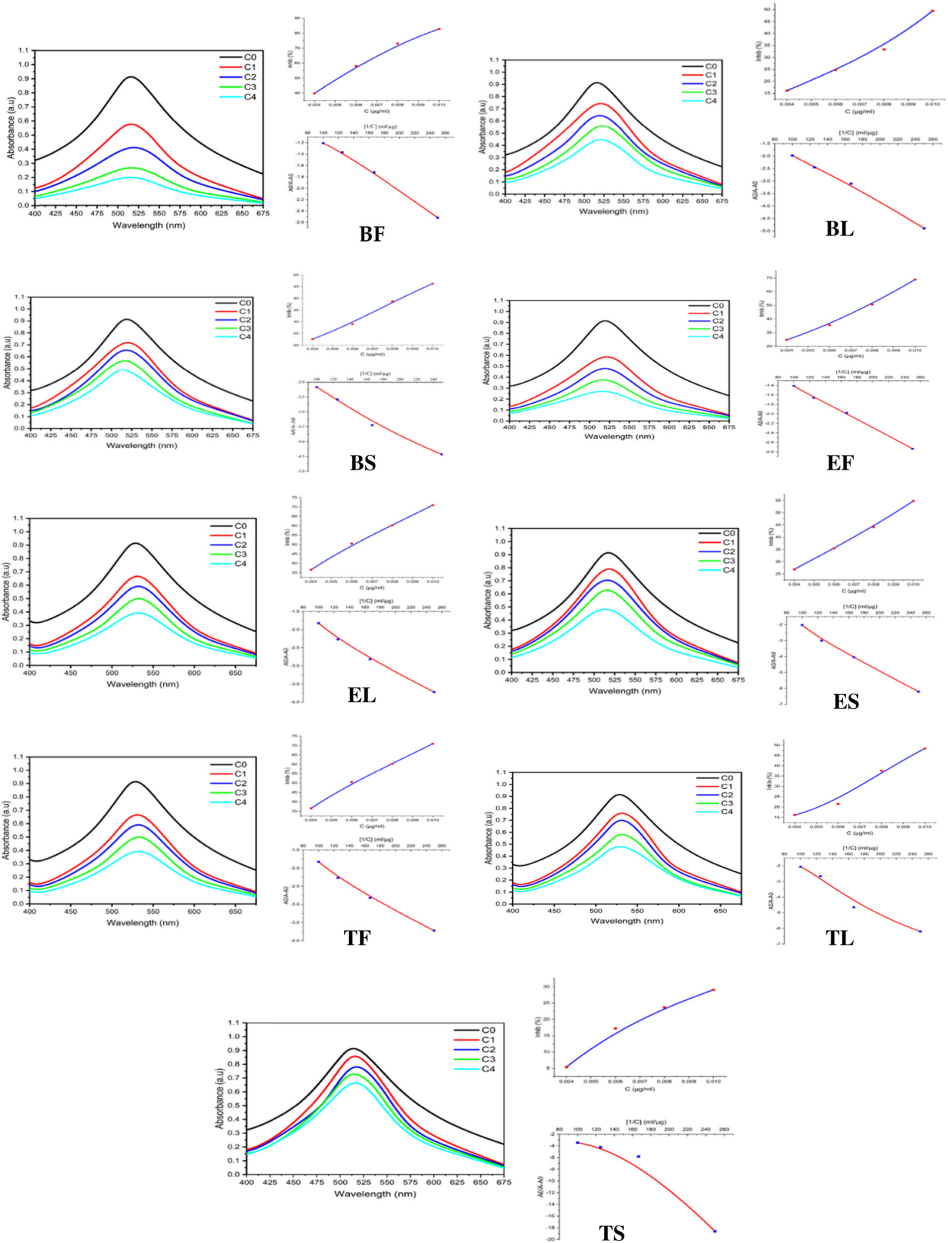
DPPH quenching activity of nine extracts from wild *S. nigrum* L. collected from three different regions in Algeria.

The ability of *S. nigrum* L. extracts to bleach the stable DPPH radical was used to evaluate their free radical-scavenging activity. All extracts quenched the DPPH-radical at all concentrations tested. The half-maximal inhibitory concentration (IC_50_) is used in the DPPH assay. The IC_50_ value is defined as the crude extract’s inhibitory concentration that scavenges 50% of ROS or inhibits the oxidation process by 50% [34]. The concentration-inhibition activity curve was used to calculate it. IC_50_ is proportional to antioxidant capacity, and a lower IC_50_ value indicates greater antioxidant activity. *S. nigrum* Tebessa fruits (TF; 0.0067), *S. nigrum* BF (0.0069), *S. nigrum* Tebessa leaves (TL; 0.0085), and *S. nigrum* EF (0.0088) had the lowest IC_50_ values (g/mL), whereas *S. nigrum* TS (0.0129) had the highest (Table 5 and Figure 6). This experiment proved that *S. nigrum* L. has antioxidant characteristics.

**Table 5:** Antioxidant activity of different *S. nigrum* L. extracts

Sample	DPPH	FRAP (mM of Fe^2+^/g)	Hemolysis assay
	IC_50_	AEAC	Protection (%)
TF	0.007 ± 0.001^ab^	257.43 ± 0.11^ab^	55.95 ± 0.14^de^
TL	0.009 ± 0.000^c^	192.73 ± 0.19^g^	39.24 ± 0.21^i^
TS	0.013 ± 0.002^de^	199.87 ± 0.28^e^	50.27 ± 0.12^g^
BF	0.006 ± 0.001^a^	211.13 ± 0.03^c^	67.84 ± 0.25^b^
BL	0.012 ± 0.002^d^	260.87 ± 0.21^a^	44.98 ± 0.33^h^
BS	0.014 ± 0.002^e^	196.83 ± 2.06^ef^	57.43 ± 0.11^d^
EF	0.008 ± 0.001^bc^	203.70 ± 0.17^d^	60.65 ± 0.67^c^
EL	0.013 ± 0.002^de^	152.10 ± 0.12^h^	28.12 ± 0.03^j^
ES	0.021 ± 0.001^f^	107.83 ± 0.21^i^	52.75 ± 0.17^f^
Ascorbic acid	0.013 ± 0.002^de^	—	72.07 ± 0.33^a^

Values with different superscript letters are statistically different.

Furthermore, the decrease in DPPH uptake in the presence of antioxidant standards can be used to calculate the binding constant and free energy, whereas the shift in wavelength values can be used to determine the mode of interaction; these findings were inspired by the study of drug molecule binding to DNA [35]. Increased reaction time between the samples and DPPH in the same solvent leads to a significant overall drop in absorbance. Based on this lack of absorbance, the binding constant has been calculated using the Benesi–Hildebrand equation, which is used to quantify an anticancer drug’s intrinsic binding constant/binding constant with DNA [36].

\[\frac{{A}_{0}}{A-{A}_{0}}=\frac{{\varepsilon }_{0}}{\varepsilon -{\varepsilon }_{0}}+\frac{{\varepsilon }_{0}}{\epsilon -{\epsilon }_{0}}\frac{1}{K\left[{\mathrm{C}}]},]\]

where *K* is the binding constant, \[{A}_{0}]\] and *A* are absorbances of DPPH in the absence and in the presence of antioxidant standards, \[{\varepsilon }_{0}]\] and *ε* are their absorption coefficients, respectively, and [C] is the concentration of antioxidant standard (Table 6).

**Table 6:** Binding constant calculation for *S. nigrum* L. different extracts

*S. nigrum*	Equation	*R* ^2^	*K* (L/mol)	∆*G* (kJ/mol)
TF	*y* = −0.015*x* − 0.293	0.88	19.53	−7.37
TL	*y* = −0.019*x* − 0.422	0.89	22.21	−7.69
TS	*y* = −0.03*x* − 1.44	0.91	48	−9.6
BF	*y* = −0.0088*x* − 0.2862	0.996	32.52	−8.63
BL	*y* = −0.01944*x* − 0.034	0.87	1.74	−1.37
BS	*y* = −0.0151*x* − 0.7292	0.982	48.29	−9.61
EF	*y* = −0.0087*x* − 0.5426	0.998	62.37	−10.25
EL	*y* = −0.0124*x* − 0.674	0.990	54.35	−9.9
ES	*y* = −0.022*x* – 0.7	0.898	31.81	−8.58

#### 3.8.2 Determination of antioxidant activity by FRAP

**Figure 7:**
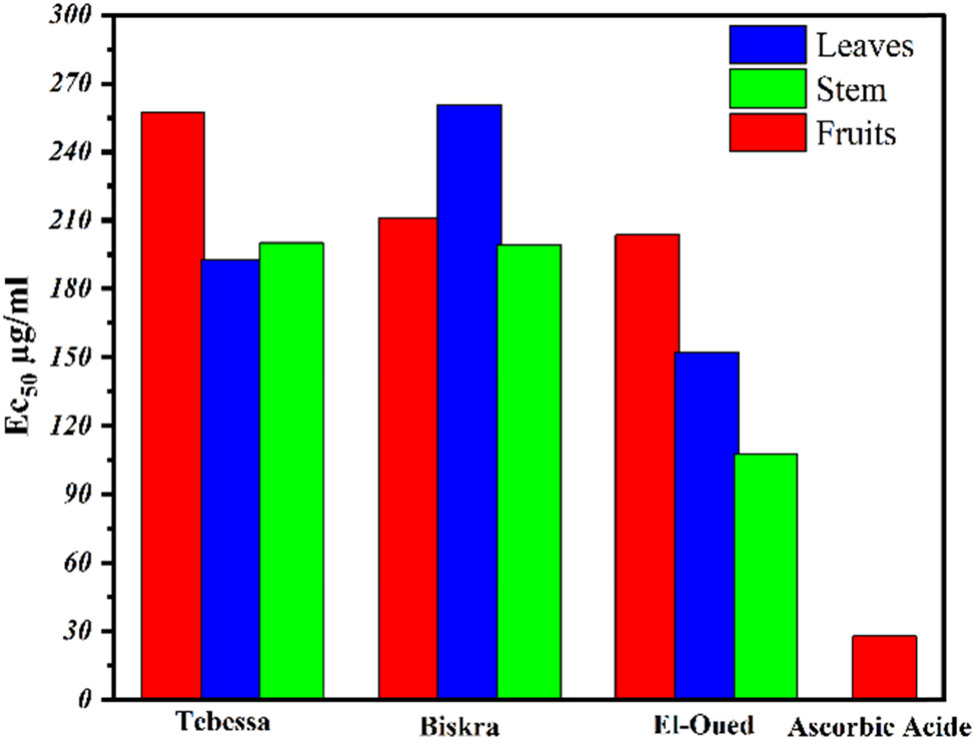
Antioxidant activity of *S. nigrum* L. extracts by FRAP.

In comparison to other assays that evaluate free radical inhibition, the FRAP test is the only one that directly analyses antioxidants (or reductants) in a sample [37]. The FRAP assay values listed in Table 5 represent the concentration of electron-donating antioxidants associated with the reduction of ferric iron (Fe^3+^) to ferrous ion (Fe^2+^) [38]. Biskra leaf extract of *S. nigrum* L. showed higher FRAP (BL; 260.9) than TF (257.34) and BF (211.18). All samples had significantly different FRAPs with values ranging from 107.85 to 260.9 mM Fe^2+^/g (dw) (Figure 7).

#### 3.8.3 PCA

**Figure 8:**
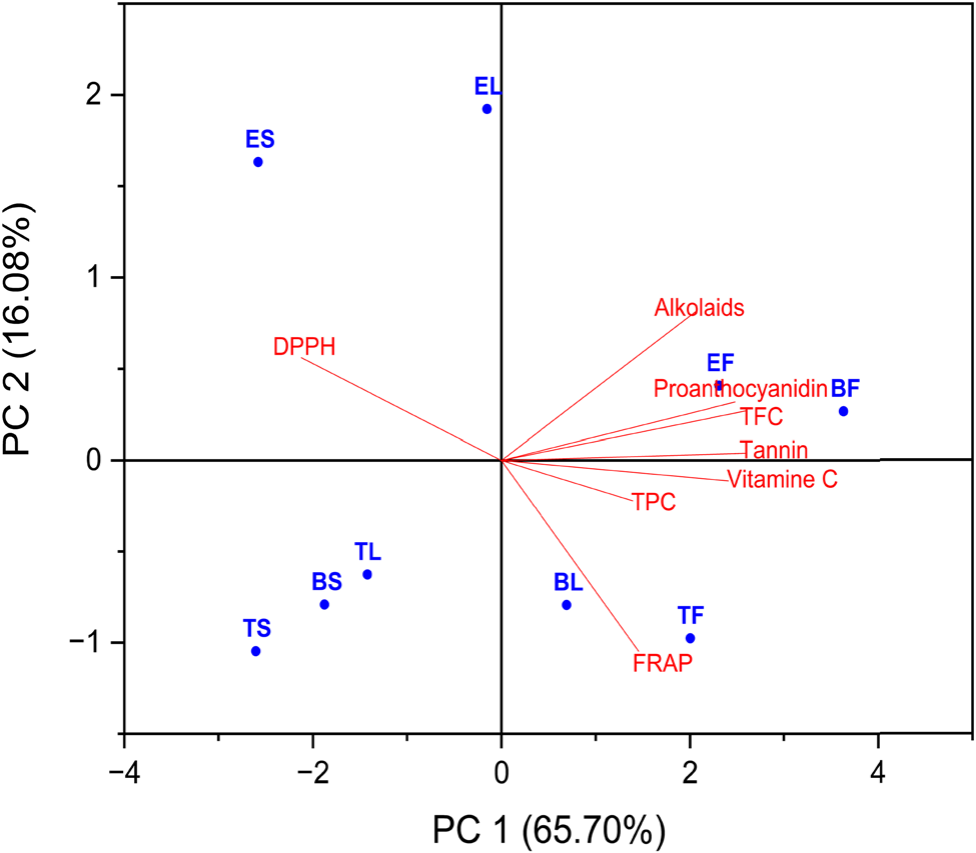
Biplot PCA of TPC, TFC, tannin, proanthocyanidins, vitamin C, and alkaloids for the nine plant extracts and antioxidant activity. The percentages PC1 and PC2 represent the largest and second-largest variances in the data on the *X*-axis and *Y*-axis, respectively.

PCA was used to get a more in-depth understanding of the connection that exists between the total content of various chemical components that were computed for each of the nine extracts and the antioxidant activity of DPPH and FRAP. The purpose of this investigation was to shed light on the relationships that exist between the nine plant extracts’ six primary constituents, which are respectively referred to as TPC, TFC, tannin, proanthocyanidins, vitamin C, and alkaloids. These are the antioxidant activity of DPPH and FRAP, which may be found in Figure 8 and Table 7, respectively.

**Table 7:** Correlation matrix between chemical content and antioxidant activity of plant extracts

	TPC	TFC	Tannin	Proanthocyanidin	Vitamin C	Alkaloids	DPPH	FRAP
TPC	1	0.40335	0.3869	0.29468	0.68767	0.24912	−0.29836	0.23047
TFC	0.40335	1	0.92158	0.90794	0.82593	0.88579	−0.71371	0.37253
Tannin	0.3869	0.92158	1	0.95028	0.76529	0.72839	−0.76237	0.51368
Proanthocyanidin	0.29468	0.90794	0.95028	1	0.73841	0.81997	−0.63373	0.34385
Vitamin C	0.68767	0.82593	0.76529	0.73841	1	0.63174	−0.60105	0.52347
Alkaloids	0.24912	0.88579	0.72839	0.81997	0.63174	1	−0.35403	−0.02724
DPPH	−0.29836	−0.71371	−0.76237	−0.63373	−0.60105	−0.35403	1	−0.70951
FRAP	0.23047	0.37253	0.51368	0.34385	0.52347	−0.02724	−0.70951	1

According to the findings of the statistical analysis that was carried out, which are included in the table, the correlation between all chemical compounds and the outcomes of the DPPH test was found to be negative. This is because the results of the antioxidant activity of the DPPH test were found to be more effective when the IC_50_ concentration was lower.

Table 8 shows that all compounds had a very strong correlation, as the concentration of the compounds increased, the concentration of IC_50_ decreased, as the correlation coefficients for the six compounds TPC, TFC, tannin, proanthocyanidins, vitamin C, and alkaloids were −0.29836, −0.71371, −0.76237, −0.63373, −0.60105, and −0.35403, respectively (as shown in Table 7).

**Table 8:** Correlation between variables (chemical content and antioxidant activity) and factors for plant extracts

	Coefficients of PC1	Coefficients of PC2
TPC	0.225	−0.14526
TFC	0.42079	0.17815
Tannin	0.41825	0.02589
Proanthocyanidin	0.40089	0.20898
Vitamin C	0.3898	−0.07314
Alkaloids	0.33449	0.53723
DPPH	−0.34301	0.36903
FRAP	0.23489	−0.68751

Table 8 illustrates the high connection that exists between chemical compounds and the outcomes of the antioxidant activity test using the FRAP test. The relationship between the two groups was found to be satisfactory and positive. This is because the mechanism of the chemicals in this antioxidant test is responsible for this positive and satisfied association. Vitamin C and tannin had the highest correlation with the FRAP test, with the correlation coefficients being 0.52347 and 0.51368, respectively. The remaining compounds had a moderate to strong correlation, with the correlation coefficients for TPC, TFC, and proanthocyanidin being 0.23047, 0.37253, and 0.34385, respectively. Vitamin C and tannin produced the strongest correlation. The correlation coefficient for the alkaloids molecule was −0.02724, which indicates that the inverse correlation was not very strong because of this.

Strong positive correlations with TFC, tannin, and proanthocyanidin were found, and the structural inputs shown in Figure 8 provided further evidence that these correlations are strong. According to the data presented in Table 8, F1 exhibited a high positive correlation with TFC (*r* = 0.41825), tannin (*r* = 0.41825), and proanthocyanidin (*r* = 0.40089).

The results of Tukey’s test (Table 5) confirm that there are large variations between the harvest area and the plant part with regard to their effectiveness in antioxidant activity, as it is clear that the BF sample is the best sample. Therefore, the Tebessa region is the best harvesting area, and the leaf part is the best part of the plant.

### 3.9 Oxidative hemolysis inhibition assay

**Figure 9:**
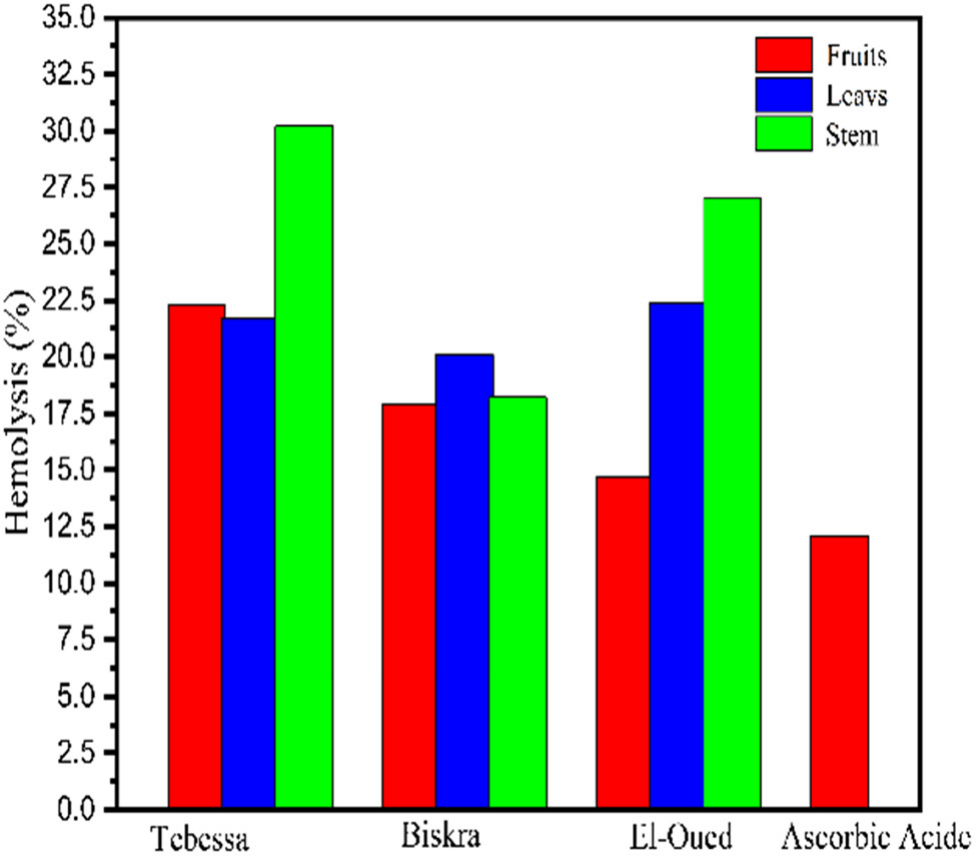
Anti-hemolytic activity of *S. nigrum* L. extracts.

Oxidative hemolysis inhibition assay is based on antioxidants’ ability to prevent free radical-induced membrane damage in erythrocytes. The benefit of this approach is that it employs peroxyl radicals as pro-oxidants and erythrocytes as oxidizable targets, resulting in results that represent biologically meaningful antioxidant micro localization and radical-scavenging activity [39]. The percentage of hemolysis was calculated as in Table 5 and the hemolysis % is inversely related to the antioxidant activity of the extract. The highest percentage indicates lower antioxidant activity, as seen in Table 5 and Figure 9, showing that EF extract of *S. nigrum* L. has the lowest percentage 14.6 which is just above the percentage of the reference antioxidant drug (ascorbic acid; 12.07), followed by TF (17.9%) and TS (18.2). The highest percentage of hemolysis was recorded by stem extract of the *S. nigrum* L. sample collected from the El-Oued region.

#### 3.9.1 PCA

**Figure 10:**
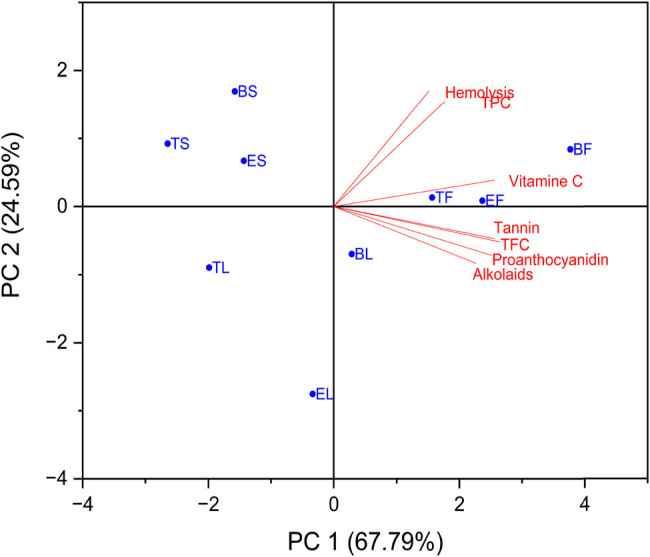
Biplot PCA of TPC, TFC, tannin, proanthocyanidins, vitamin C, and alkaloids for the nine plant extracts and hemolysis test. The percentages of PC1 and PC2 represent the largest and second largest variance in the data on the *X*-axis and *Y*-axis, respectively.

PCA was used to acquire a more profound comprehension of the connection that exists between the total content of various chemical components that were computed for each of the nine extracts and the hemolysis test. By examining the nine plant extracts and the hemolysis test, this investigation aimed to shed light on the relationships that exist between the six primary components, which are as follows: TPC, TFC, tannin, proanthocyanidins, vitamin C, and alkaloids. Figure 10 and Table 9 present the results of this investigation. According to the findings of the statistical analysis that was carried out, which are presented in the table, the correlation between all of the chemical compounds and the outcomes of the hemolysis test was a strong and positive correlation. This indicates that the percentage of protection increases in proportion to the concentration of chemical compounds. The correlation coefficient for TPC was 0.97698, which indicates that the concentration of TPC plays a very significant influence on the protection rate. Table 9 demonstrates that the correlation between TPC and the protection rate was extremely high, and it was very near to 1. The correlation coefficient for vitamin C was 0.62896, and the correlation coefficient for alkaloids was 0.09513. TFC, tannin, and proanthocyanidin each had a correlation value of 0.29827, 0.3147, and 0.20262, respectively, falling somewhere in the middle of the moderate to strong range. The coefficient of correlation for alkaloids was 0.09513, which indicates that the connection was very weak despite the fact that it was positive. A significant positive connection with TPC was found to exist, and this finding is supported by the structural inputs shown in Figure 10. PC1 was found to have a strong positive relationship with TFC (*r* = 0.28985), and PC2 was found to have a strong positive relationship with TFC (*r* = 0.57856). This information is shown in Table 10.

**Table 9:** Correlation matrix between chemical content and hemolysis test of plant extracts

	TPC	TFC	Tannin	Proanthocyanidin	Vitamin C	Alkaloids	Hemolysis
TPC	1	0.40335	0.3869	0.29468	0.68767	0.24912	0.97698
TFC	0.40335	1	0.92158	0.90794	0.82593	0.88579	0.29827
Tannin	0.3869	0.92158	1	0.95028	0.76529	0.72839	0.3147
Proanthocyanidin	0.29468	0.90794	0.95028	1	0.73841	0.81997	0.20262
Vitamin C	0.68767	0.82593	0.76529	0.73841	1	0.63174	0.62896
Alkaloids	0.24912	0.88579	0.72839	0.81997	0.63174	1	0.09513
Hemolysis	0.97698	0.29827	0.3147	0.20262	0.62896	0.09513	1

**Table 10:** Correlation between variables (chemical content and hemolysis test) and factors for plant extracts

	Coefficients of PC1	Coefficients of PC2
TPC	0.28985	0.57856
TFC	0.43579	−0.195
Tannin	0.42136	−0.17682
Proanthocyanidin	0.41333	−0.26925
Vitamin C	0.42136	0.14588
Alkaloids	0.37215	−0.31254
Hemolysis	0.24829	0.63602

The Tukey test results (Table 5 and Figure 9) confirm that there are significant differences between the harvest area and the plant part with regard to their effectiveness in protecting red blood cells, and it is also clear that the BF sample is the best sample. Therefore, the Tebessa region is considered the best area for harvesting, and the leafy part is the best part of the plant

## 4 Correlation between phenolic phytochemicals of *S. nigrum* and it’s antioxidant activity via In-sillico docking studies

The aim of this part of study was to investigate the correlation of the phenolic chemical composition of *S. nigrum* L. with their antioxidant activity. Fifteen polyphenolic compounds were previously reported as the major isolated and identified compound from *S. nigrum* L. methanol extract as reported by Chen et al. [40], which are listed in Table S1. Based on the physicochemical and ADME properties of major phenolic compounds reports in *S. nigrum* L., *in silico* docking study was performed on only ten compounds to determine the potential antioxidant mechanism of action.

### 4.1 *In silico* evaluation of physicochemical and ADME properties of major phenolic compound reports in *S. nigrum* L.

A computational study of major phenolic compounds [1–14] presented in the *S. nigrum* L. plant was performed to evaluate physicochemical and ADME properties using SwissADME [41]. With respect to physicochemical properties (Table 11), all the reported compounds have zero violation of the Lipinski’s rule for oral drugs, except for compounds 2–5 and 14, which have two violations and more. So, we pursue studies on the other ten compounds (1, 6–13, and 15). All the topological polar surface areas (TPSA) of the chosen ten compounds are less than 131.36 A^0 2^. Additionally, absorption (% ABS) was estimated by using the equation% ABS = 109 – (0.345 × TPSA) [42] and found that the calculated% ABS of those compounds ranged between 63.68 and 89.15%, demonstrating that those compounds may have the required cell membrane permeability and bioavailability. All ten compounds have rotatable bonds between 1 and 2, which indicate molecular flexibility to their biotarget. Regarding pharmacokinetics, it was found that the chosen ten compounds have high gastrointestinal absorption and have no permeation to the blood–brain barrier except for 4-hydroxybenzoic acid (compound 11) and 4-hydroxycinnamic acid (compound 15), which have high GIT absorption and can pass blood–brain barrier. Thus, ensuring that almost all compounds will have low to no central nervous system side effects. Bioavailability is an index of the amount of drug present in the plasma and is considered the most crucial factor affecting absorption. Interestingly, all the compounds were found to have high bioavailability scores ranging from 0.55 to 0.85.

**Table 11:** Physicochemical properties of major phenolic compounds previously isolated from *Solanum nigrum* L. based on Lipinskí’s rule of five, TPSA, % ABS, and number of rotatable bonds

No.	Comp. name	HBD	HBA	MlogP	MW	Lipinski’s violations	TPSA	% ABS	No. of rot. bonds
1	Quercetin	5	7	−0.56	302.24	0	131.36	63.68	1
2	Quercitrin	7	11	−1.84	448.38	2	190.28	43.35	3
3	Isoquercitrin	8	12	−2.59	464.38	2	210.51	36.37	4
4	Quercetin-3-gentiobioside	11	17	−4.62	626.52	3	289.66	9.06	7
5	6-Hydroxyluteolin 7-sophoroside	11	17	−4.62	626.52	3	289.66	9.06	7
6	Kaempferol	4	6	−0.03	286.24	0	111.13	70.66	1
7	Gallic acid	4	5	−0.16	170.18	0	97.99	75.19	1
8	2,4-Dihydroxybenzoic acid	3	4	0.4	154.12	0	77.76	82.17	1
9	Protocatechuic acid	3	4	0.4	154.12	0	77.76	82.17	1
10	Vanillic acid	2	4	0.74	168.15	0	66.76	85.96	2
11	4-Hydroxybenzoic acid	2	3	0.99	138.12	0	57.53	89.15	1
12	2,5-Dihydroxybenzoic acid (gentisic acid)	3	4	0.4	154.12	0	77.76	82.17	1
13	Caffeic acid	3	4	0.7	180.16	0	77.76	82.17	2
14	Chlorogenic acid	6	9	−1.05	354.31	1	164.75	52.16	5
15	4-Hydroxycinnamic acid	2	3	1.28	164.16	0	57.53	89.15	2

### 4.2 *In silico* antioxidant docking study

**Figure 11:**
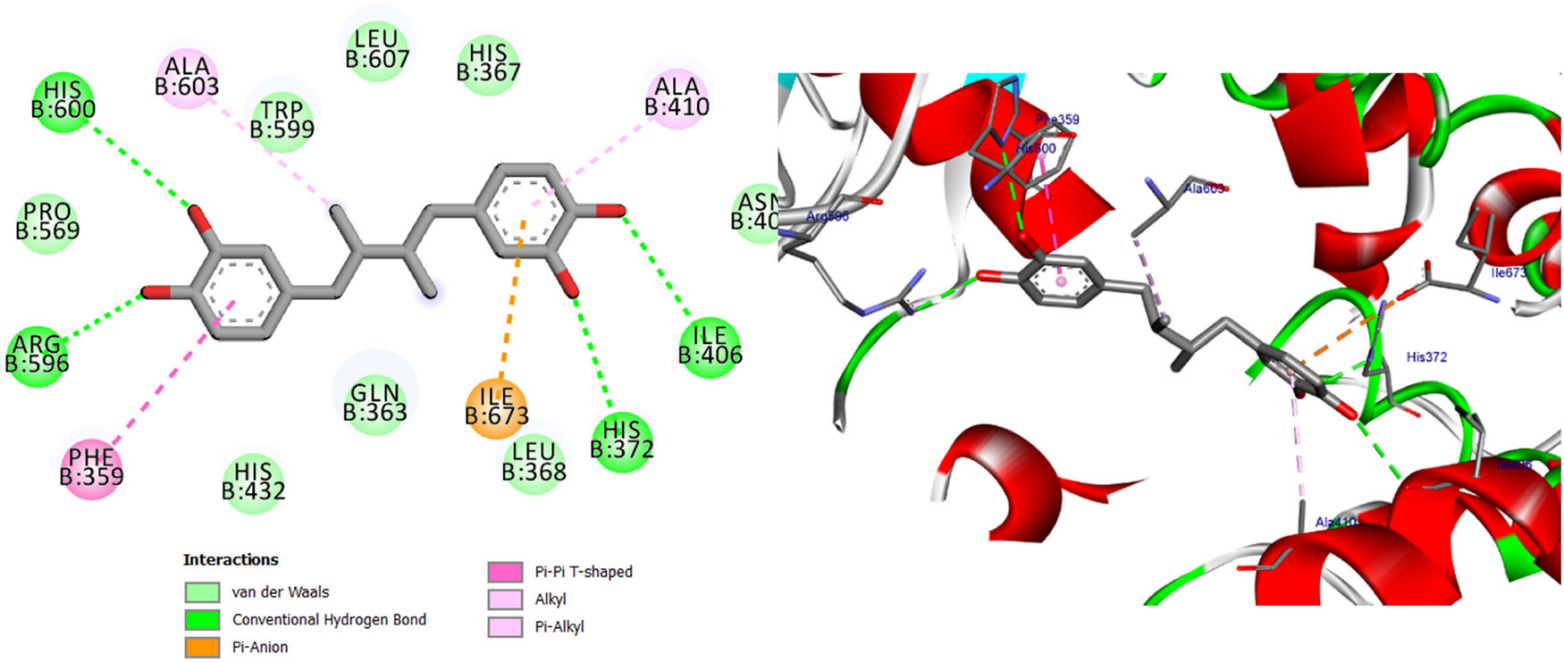
Two- and three-dimensional protein/ligand interaction between bioactive compound NDGA, and arachidonate-5-LOX (ALOX5, PDB: 6n2w).

**Figure 12:**
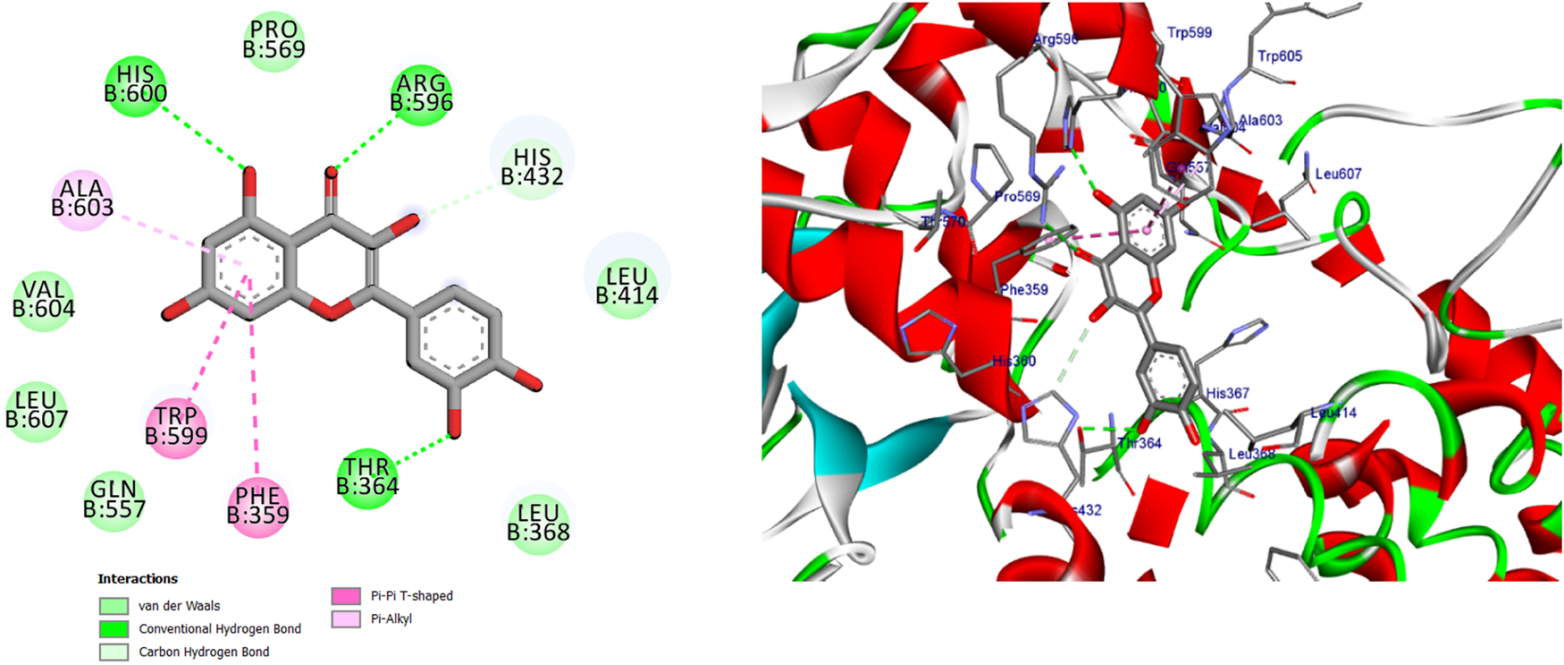
Two- and three-dimensional protein/ligand interaction between bioactive compound Quercetin, and arachidonate-5-LOX (ALOX5, PDB: 6n2w).

**Figure 13:**
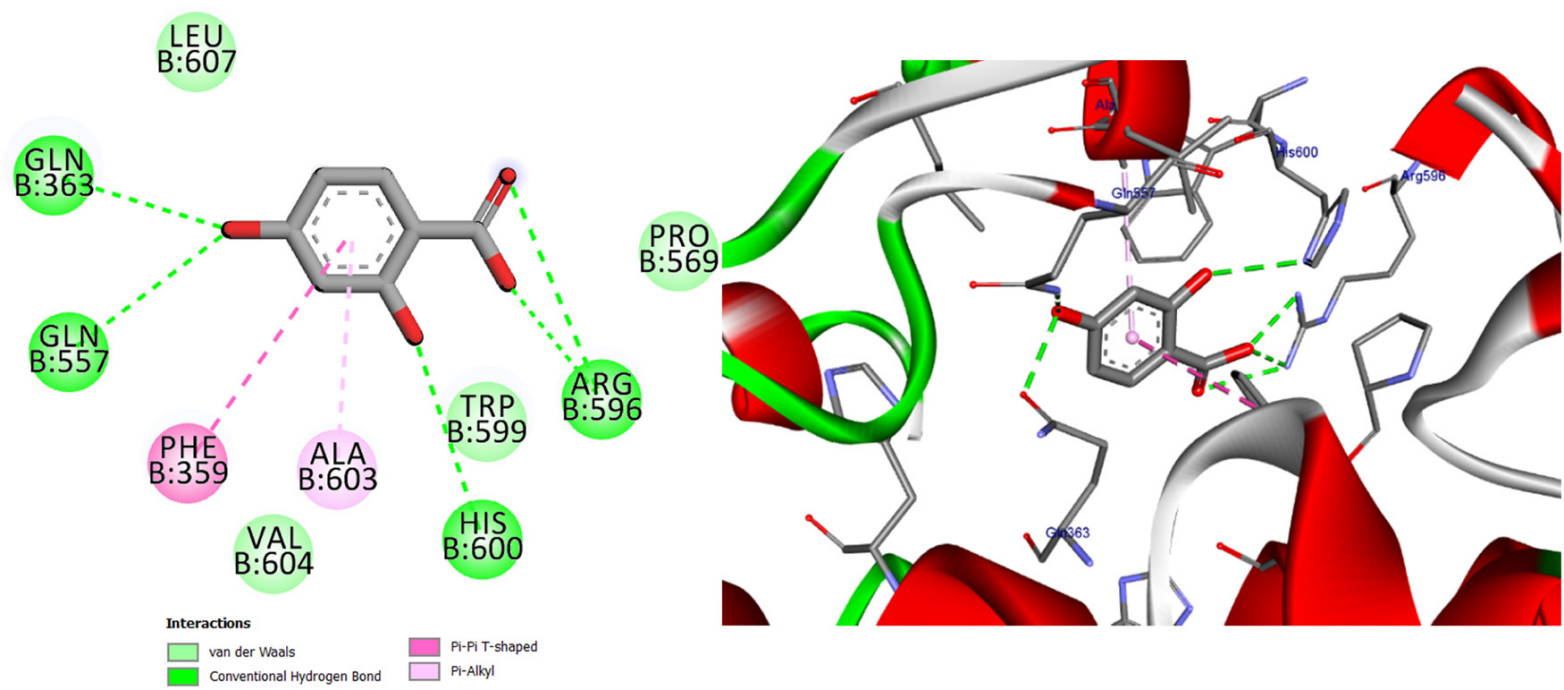
Two- and three-dimensional protein/ligand interaction between 2,4-dihydroxybenzoic acid and arachidonate-5-LOX (ALOX5, PDB: 6n2w).

**Figure 14:**
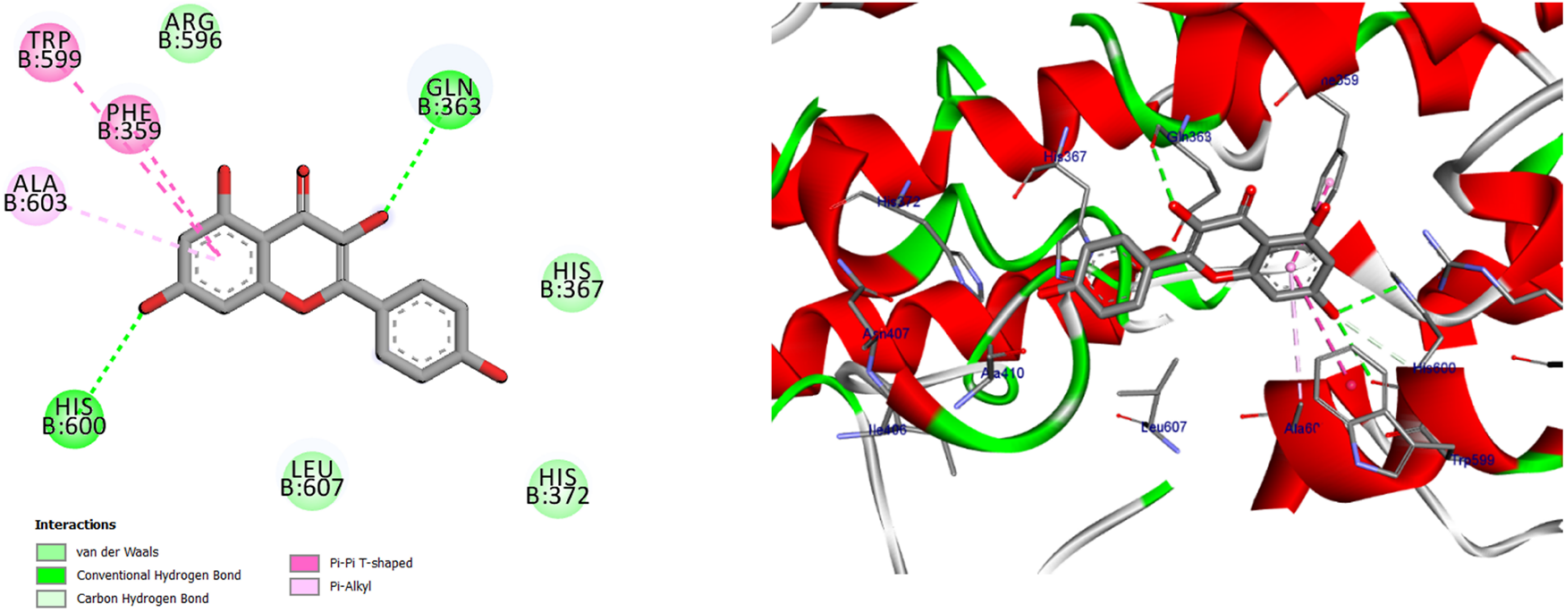
Two- and three-dimensional protein/ligand interaction between kaempferol and arachidonate-5-LOX (ALOX5, PDB: 6n2w).

**Figure 15:**
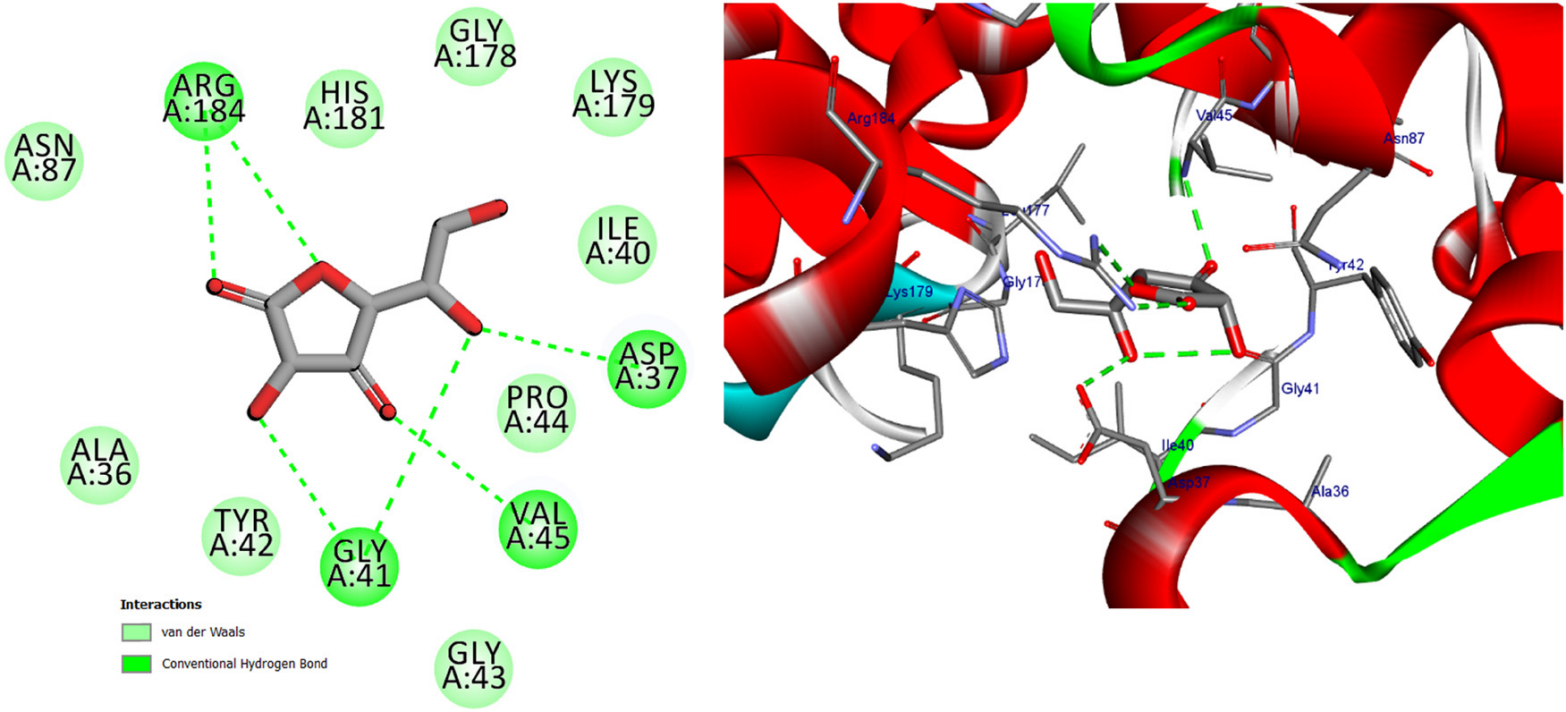
Two- and three-dimensional protein/ligand interaction between ascorbic acid and cytochrome *c* peroxidase (PDB: 2x08).

**Figure 16:**
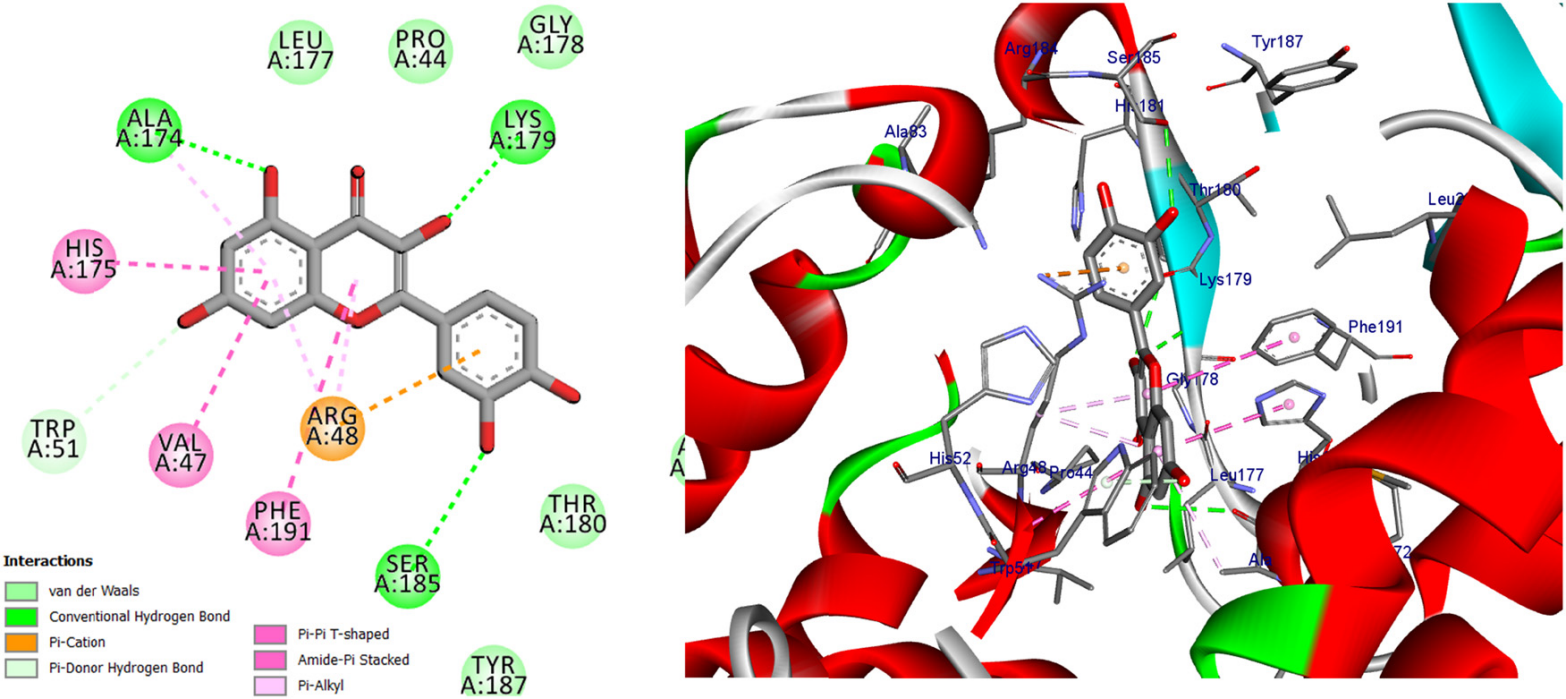
Two- and three-dimensional protein/ligand interaction between quercetin and cytochrome *c* peroxidase (PDB: 2x08).

**Figure 17:**
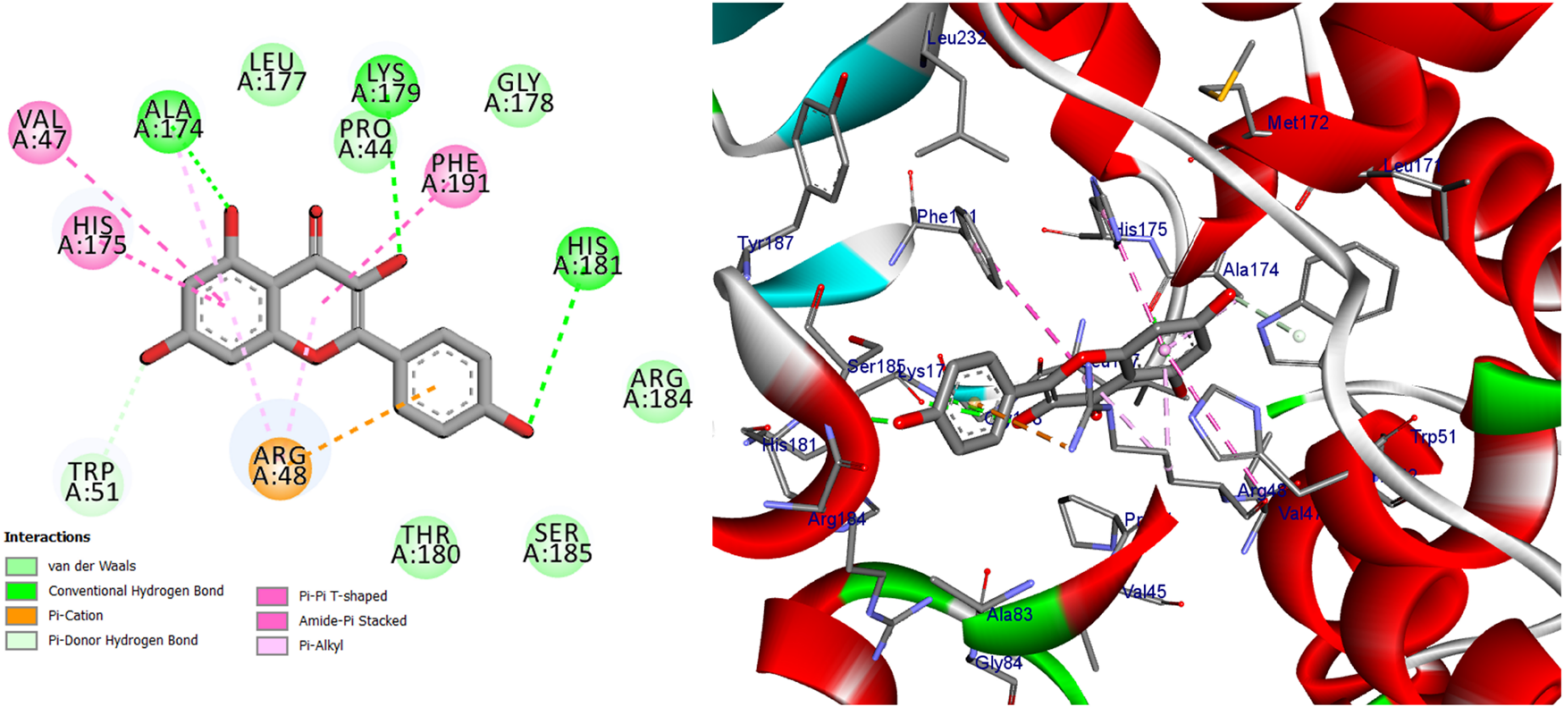
Two- and three-dimensional protein/ligand interaction between kaempferol and cytochrome *c* peroxidase (PDB: 2x08).

**Figure 18:**
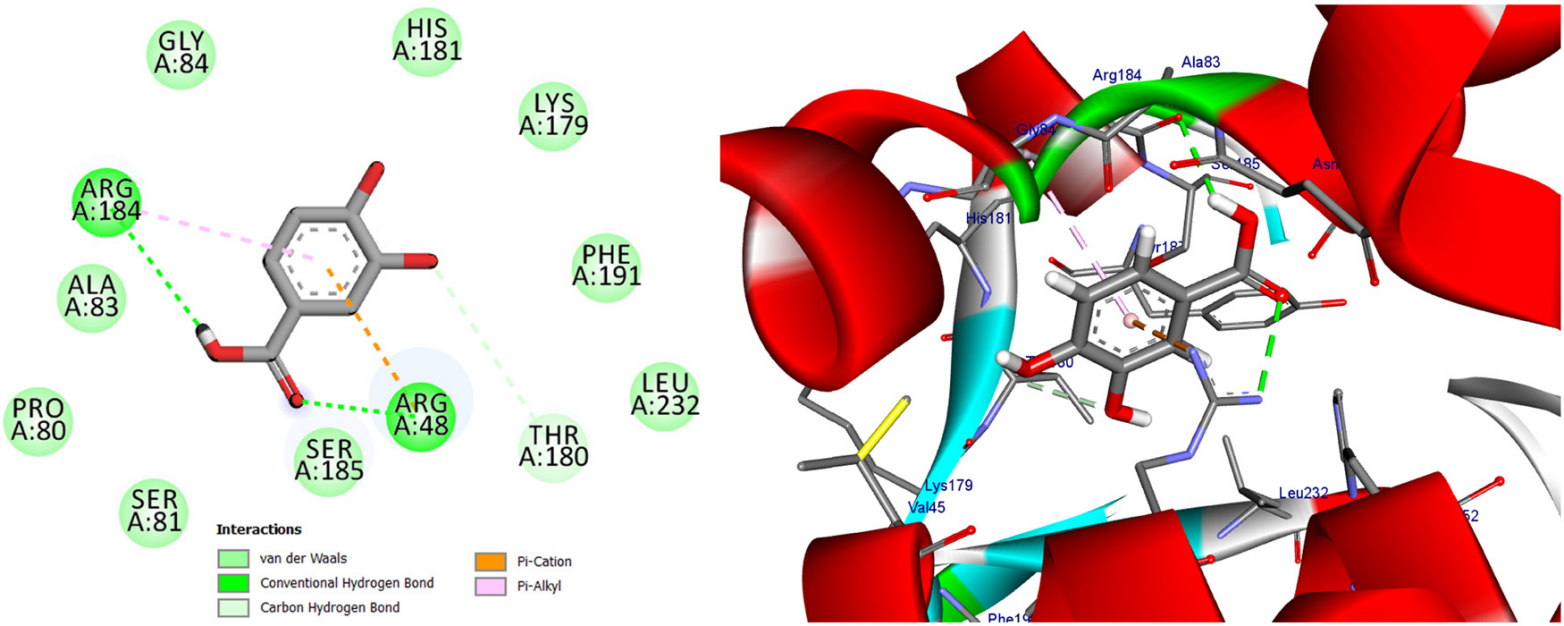
Two- and three-dimensional protein/ligand interaction between protocatechuic acid and cytochrome *c* peroxidase (PDB: 2x08).

Docking seeks to appropriately assess the strength of binding by accurately predicting a ligand’s shape inside the confines of a binding pocket. The ten bioactive compounds quercetin, kaempferol, gallic acid, 2,4-dihydroxybenzoic acid, protocatechuic acid, vanillic acid, 4-hydroxybenzoic acid, 2,5-dihydroxybenzoic acid (gentisic acid), caffeic acid, and 4-hydroxycinnamic acid were docked with selected proteins, arachidonate-5-lipoxygenase (5-LOX) (PDB: 6n2w) [3] and cytochrome *c* peroxidase (PDB: 2x08), to check their affinity with the binding sites of these proteins for the possible mechanism of action. According to the docking scores, quercetin and kaempferol of *S. nigrum* L. may have a crucial role as antioxidant. Our results are shown in Table 12, Tables S2 and S3, Figures 11–18, and Figures S1 and S2.

**Table 12:** Docking scores and binding interactions of selected phenolic compounds

Com. no.	LOX			Peroxidase		
	Docking score (kcal/mol)	Type of interaction (interacting amino acids)	Distance (A^0^)	Docking score (kcal/mol)	Type of interaction (interacting amino acids)	Distance (A^0^)
NDGA	−11.01	Hydrogen bond^1^(His372)	2.93			
		Hydrogen bond^1^(Arg596)	3.08			
		Hydrogen bond^1^(His600)	3.10			
		Hydrogen bond^1^(Ile406)	3.37			
		Pi-anion(Ile673)	4.68			
		Pi–Pi T-shaped(Phe359)	5.11			
		Alkyl(Ala603)	4.39			
		Pi-Alkyl(Ala410)	4.63			
Ascorbic acid				−9.71	Hydrogen bond^1^(Val45)	2.77
					Hydrogen bond^1^(Arg184)	3.32
					Hydrogen bond^1^(Arg184)	2.79
					Hydrogen bond^1^(Gly41)	2.69
					Hydrogen bond^1^(Asp37)	2.59
					Hydrogen bond^1^(Gly41)	3.34
Quercetin	−11.86	Hydrogen bond^1^(Thr364)	2.95	−14.82	Hydrogen bond^1^(Lys179)	3.28
		Hydrogen bond^1^(Arg596)	2.99		Hydrogen bond^1^(Ser185)	2.91
		Hydrogen bond^1^(His600)	2.91		Hydrogen bond^1^(Ala174)	3.33
		Hydrogen bond^2^(His432)	3.70		Hydrogen bond^1^(Lys179)	3.20
		Pi–Pi T-shaped(Phe359)	5.30		Pi-cation(Arg48)	4.23
		Pi–Pi T-shaped(Trp599)	5.36		Hydrogen Bond^3^(Trp51)	3.35
		Pi-Alkyl(Ala603)	4.37		Pi–Pi T-shaped(His175)	4.48
					Pi–Pi T-shaped(Phe191	5.57
					Amide-Pi stacked(Val47/Arg48)	5.20
					Pi-alkyl(Arg48)	3.96
					Pi-alkyl(Arg48)	3.98
					Pi-alkyl(Ala174)	4.49
Kaempferol	−11.11	Hydrogen bond^1^(His600)	3.36	−14.27	Hydrogen bond^1^(Lys179)	3.26
		Hydrogen bond^1^(His600)	2.94		Hydrogen bond^1^(His181)	3.34
		Hydrogen bond^1^(Gln363)	3.02		Hydrogen bond^1^(Ala174)	3.34
		Hydrogen bond^2^(His600)	3.64		Hydrogen bond^1^(Lys179)	3.07
		Pi–Pi T-shaped(Phe359)	5.10		Pi-cation(Arg48)	4.25
		Pi–Pi T-shaped(Trp599)	5.50		Hydrogen bond^3^(Trp51)	3.36
		Pi-alkyl(Ala603)	5.02		Pi–Pi T-shaped(His175)	4.45
					Pi–Pi T-shaped(Phe191)	5.53
					Amide-Pi stacked(Val47/Arg48)	5.25
					Pi-alkyl(Arg48)	3.97
					Pi-alkyl(Arg48)	3.96
					Pi-alkyl(Ala174)	4.57
Gallic acid	−10.72	Hydrogen bond^1^(Arg596)	3.06	−10.56	Hydrogen bond^1^(Arg48)	3.16
		Hydrogen bond^1^(Arg596)	3.04		Hydrogen bond^1^(Trp51)	3.07
		Hydrogen Bond^1^(Gln363)	2.98		Hydrogen bond^1^(Lys179)	3.26
		Pi–Pi T-shaped(Phe359)	5.37		Hydrogen bond^1^(Lys179)	2.98
		Pi-Alkyl(Ala603)	5.14		Hydrogen bond^1^(Lys179)	3.07
					Hydrogen bond^1^(Pro44))	3.24
					Pi–Pi T-shaped(Phe191)	5.45
					Pi-Alkyl(Arg48)	4.02
2,4-Dihydroxybenzoic acid	−11.15	Hydrogen bond^1^(Gln557)	3.10	−10.98	Hydrogen bond^1^(Lys179)	2.96
		Hydrogen bond^1^(Arg596)	3.11		Hydrogen bond^1^(Arg184)	3.00
		Hydrogen bond^1^(Arg596)	3.34		Hydrogen bond^1^(Arg184)	3.20
		Hydrogen bond^1^(Arg596)	3.17		Hydrogen bond^1^(Gly41))	2.50
		Hydrogen bond^1^(Gln363)	2.86		Pi-Cation(His181)	4.74
		Hydrogen bond^1^(His600)	3.12		Pi–Pi T-shaped(His181)	4.79
		Pi–Pi T-shaped(Phe359)	5.26		Pi-Alkyl(Pro44)	4.63
		Pi-Alkyl(Ala603)	4.90		Pi-Alkyl(Val45)	4.55
Protocatechuic acid	−10.36	Hydrogen bond^1^(Arg596)	3.01	−12.10	Hydrogen bond^1^(Arg48)	2.92
		Hydrogen bond^1^(Arg596)	3.04		Hydrogen bond^1^(Arg184)	2.71
		Hydrogen bond^1^(Gln359)	2.89		Hydrogen Bond^2^(Thr180)	3.79
		Pi–Pi T-shaped(Phe359)	5.23		Pi-cation(Arg48)	2.93
		Pi-alkyl(Ala603)	5.15		Pi-alkyl(Arg184)	4.70
Vanillic acid	−9.19	Hydrogen bond^1^(Arg596)	3.12	−10.81	Hydrogen bond^1^(His181)	2.91
		Hydrogen bond^1^(Arg596)	3.19		Hydrogen bond^1^(Ala83)	2.73
		Hydrogen bond^1^(His600)	2.91		Hydrogen bond^2^(Arg184)	3.27
		Hydrogen bond^1^(Gln363)	3.00		Hydrogen bond^4^(Arg184)	3.67
		Hydrogen bond^2^(Arg596)	3.38		Pi-Alkyl(Arg48)	4.90
		Pi–Pi T-shaped(Phe359)	5.41		Pi-Alkyl(Arg184)	5.46
		Pi-Alkyl(Ala603)	4.75			
4-Hydroxybenzoic acid	−11.10	Hydrogen bond^1^(Gln557)	3.24	−9.92	Hydrogen bond^1^(Lys179)	2.96
		Hydrogen bond^1^(Arg596)	2.96		Hydrogen bond^1^(Arg184)	3.02
		Hydrogen bond^1^(Arg596)	3.20		Hydrogen bond^1^(Arg184)	3.20
		Hydrogen bond^1^(His600)	2.89		Hydrogen bond^1^(Gly41)	3.13
		Hydrogen bond^1^(Gln363)	2.80		Hydrogen bond^1^(Lys179)	3.10
		Pi–Pi T-shaped(Phe359)	5.12		Pi-cation(His181)	4.71
		Pi-Alkyl(Ala603)	4.59		Pi–Pi T-shaped(His181)	4.77
					Pi-Alkyl(Pro44)	4.62
					Pi-Alkyl(Val45)	4.61
2,5-Dihydroxybenzoic acid (gentisic acid)	−10.74	Hydrogen bond^1^(Arg596)	3.20	−11.49	Hydrogen bond^1^(His181)	2.79
		Hydrogen bond^1^(Arg596)	3.09		Hydrogen bond^1^(Lys179)	2.58
		Hydrogen bond^1^(Gln363)	3.00		Hydrogen bond^1^(Ala83)	2.51
		Hydrogen bond^1^(His600)	3.02		Hydrogen bond^1^(Arg184)	3.37
		Pi–Pi T-shaped(Phe359)	5.34		Hydrogen bond^1^(Ala83)	2.51
		Pi-Alkyl(Ala603)	4.65		Hydrogen bond^2^(Thr180)	3.16
					Hydrogen bond^2^(Ser185)	3.22
					Hydrogen bond^4^(Arg184)	3.93
					Pi-Alkyl(Arg184)	4.26
Caffeic acid	−10.75	Hydrogen bond^1^(Thr364)	3.29	−11.18	Pi-Cation(His175)	4.81
		Hydrogen bond^1^(his432)	3.06		Pi–Pi T-shaped(His175)	3.86
		Hydrogen bond^1^(Arg596)	3.26		Pi-Alkyl(Arg48)	5.34
		Hydrogen bond^1^(Arg596)	3.38			
		Hydrogen bond^1^(His600)	3.15			
		Pi–Pi T-shaped (Phe359)	5.57			
4-Hydroxycinnamic acid	−8.69	Hydrogen bond^1^(His432)	3.18	−10.90	Hydrogen bond^1^(Ser185)	2.99
		Hydrogen bond^1^(Gln363)	2.92		Hydrogen bond^1^(Pro44)	3.10
		Hydrogen bond^1^(His600)	3.35		Hydrogen bond^2^(Arg184)	3.77
					Pi-Alkyl (Agr48)	4.08

^1^Conventional hydrogen bond.

^2^Carbon hydrogen bond.

^3^Pi-donor hydrogen bond.

^4^Pi-cation; Pi-donor hydrogen bond.

#### 4.2.1 Docking into LOX

LOXs are a class of enzymes responsible for facilitating the oxidation process of polyunsaturated fatty acids and lipids that possess a *cis*, *cis*-l.4-pentadiene structure [43]. LOXs are enzymes involved in the oxidation of lipids, and their role in the generation of oxidized lipids within atherosclerotic lesions is widely acknowledged. The enzyme known as arachidonate 5-LOX is of significant importance in the process of leukotriene production. The excessive activation of the 5-LOX pathway leads to the production of an abundance of leukotrienes and lipoxins. These bioactive molecules possess high cytotoxic properties and play a significant role in various pathophysiological conditions such as cancer, psoriasis, and atherosclerosis [44].

The involvement of oxidative stress has been suggested in the pathophysiology of various diseases. The inhibition of 5-LOX has been shown to provide cellular protection against oxidative stress [45]. The active site of 5-LOX consists of certain amino acid residues, namely His367, His372, His550, Leu673, Gln363, Ala410, His432, Gln557, and His600 [46]. The co-crystallized ligand NDGA was re-docked into its corresponding enzyme, resulting in a measured root mean square deviation (RMSD) of 0.62 Å between the docked and co-crystallized ligand. This finding indicates the validity of the docking approach employed. The ligand NDGA, which was co-crystalized, exhibits a docking score of −11.11 kcal/mol. It forms hydrogen bonds with amino acid residues His372, Arg596, His600, and Ile406. Additionally, it engaged in four hydrophobic interactions with Ile673, Phe359, Ala603, and Ala410, as depicted in Figure 11. All the compounds that were chosen for this study exhibit binding within the active site of 5-LOX in a manner similar to that of the co-crystal ligand, NDGA, as depicted in Figure 1. In addition, the binding energy of the ten compounds exhibited a range of −8.69 to −11.86 kcal/mol. The majority of compounds exhibited a Pi–Pi T-shaped link with Phe359, a Pi-Alkyl bond with Ala603, and one or more hydrogen bonds with Gln363, Arg596, and His600, as indicated in Table 12 and Table S2. We conducted a search to identify the optimal conformations that exhibited the lowest binding energy (∆*G*, kcal/mol) for quercetin and 2,4-dihydroxybenzoic acid. In Table 12, it can be observed that quercetin had a binding energy of −11.86 kcal/mol, whereas 2,4-dihydroxybenzoic acid displayed a binding value of −11.15 kcal/mol. Both compounds exhibited hydrogen bond interactions with specific amino acid residues, namely Arg596 and His600, in the inhibition of 5-LOX. For quercetin, the hydrogen bond distances were measured at 2.99 Å for Arg596 and 2.91 Å for His600. In the case of 2,4-dihydroxybenzoic acid, the hydrogen bond distances were recorded at 3.11, 3.34, and 3.34 Å for Arg596 and 3.12 Å for His600. These findings are depicted in Figures 12 and 14. Furthermore, it was seen that quercetin formed two hydrogen connections with Thr364 and His432, as well as a Pi–Pi T-shaped interaction with Trp599. In contrast, it was shown that 2,4-dihydroxybenzoic acid established a total of two hydrogen bonds with Gln557 and Gln363. Kaempferol had a docking score equivalent to that of NDGA (−11.11 kcal/mol). Its stabilization was facilitated by three hydrogen bonding interactions involving the 4-hydroxyl group and the amino acid His600. In addition, it was shown that the compound is known as Kaempferol, while in the form of quercitrin, exhibited the formation of two Pi–Pi T-shaped connections with amino acid residues Phe359 and Trp599, as well as a Pi-Alkyl link with Ala603 (as depicted in Figure 14).

#### 4.2.2 Docking into cytochrome *c* peroxidase enzyme

In addition to its primary function, cytochrome *c* peroxidase has the ability to facilitate the oxidation of several substrate molecules in the presence of hydrogen peroxide (H_2_O_2_). The peroxidase activity has garnered significant attention owing to its involvement in the process of apoptosis [47]. The binding scores of our candidates (−9.92, −14.82 kcal/mol) were found to be highly promising when compared to the binding score of ascorbic acid (−9.71 kcal/mol), as presented in Table 12. Based on the obtained data, it may be inferred that the aforementioned compounds exhibit stabilization within the binding pocket of cytochrome *c* peroxidase. The RMSD between the original and docked poses of ascorbic acid in the peroxidase enzyme (PDB: 2x08) was determined to be 0.20 Å. Furthermore, the stabilization of ascorbic acid occurred within the binding pocket of cytochrome *c* peroxidase through the formation of six hydrogen bonds with specific amino acid residues. These residues include Val45 (at a distance of 2.77 Å), Gly41 (at distances of 2.69 and 3.34 Å), Asp37 (at a distance of 2.59 Å), and Arg184 (at distances of 3.32 and 2.79 Å) (refer to Figure 15). The stabilization of candidates inside the binding pocket was achieved through their interaction with essential amino acids, as illustrated in Table 12 and Table S3. The docking scores of quercetin and kaempferol were found to be −14.82 and −14.27 kcal/mol, respectively. These compounds exhibited strong binding affinities to important amino acids, namely Lys179, Ala174, and Trp51, through the formation of five hydrogen bonds. Additionally, they formed three Pi-Alkyl bonds with Arg48 and Ala174, and two Pi–Pi T-shaped bonds with His175 and Phe191. Furthermore, a Pi-cation bond was observed between these compounds and Arg48, with a distance of 4.23 Å. Additionally, it was observed that quercetin and kaempferol exhibited an Amid-Pi stacking interaction with Val47 and Arg48 at a distance of 5.20 Å, as depicted in Figures 16 and 17. The binding of protocatechuic acid occurred by the formation of three hydrogen bonds with Arg48, Arg184, and Thr180, at distances of 2.92, 2.71, and 3.79 Å, respectively. Further, it was observed that protocatechuic acid formed a Pi-cation bond with Arg48 at a distance of 2.93 Å and a Pi-alkyl bond with Arg184 at a distance of 4.70 Å, as depicted in Figure 18. The binding energy value of gentisic acid was determined to be −11.49 kcal/mol. This compound was found to be bound to crucial amino acid residues, including Lys179, Arg184, His181, Ala83, Thr180, and Ser185, through a total of eight hydrogen bonds, as indicated in Table S3. Ultimately, the majority of the compounds were bound by one or more H-bonds and/or hydrophobic bonds to the vital amino acids Arg48, Lys179, and Arg184. The receptor interactions of substances with the (2×08) protein were analyzed in both two-dimensional (2D) and three-dimensional (3D) formats. The results of these analyses are shown in Table 12. Additionally, for more comprehensive examination, the corresponding figures illustrating these interactions may be found in the Supplementary Materials, specifically in Table S3.

## 5 Conclusions

In conclusion, this study highlights the potential of *S. nigrum* Linn. as a valuable natural source of antioxidants. The comparative phytochemical profiling revealed the presence of various bioactive components, including steroidal saponins, alkaloids, flavonoids, phenols, and proanthocyanidins, in the Algerian *S. nigrum* samples. The assessment of antioxidant activity through DPPH, FRAP, and oxidative hemolysis inhibition assays demonstrated the substantial antioxidant potential of the three samples. These findings support the notion that *S. nigrum* can effectively scavenge free radicals and mitigate oxidative stress.

Furthermore, the *in silico* molecular docking analysis provided insights into the potential mechanism of action of *S. nigrum*’s antioxidant activity. The docking scores indicated a significant affinity for the bioactive compounds, particularly quercetin, and kaempferol, with the binding sites of selected proteins, arachidonate-5-lipoxygenase, and cytochrome *c* peroxidase. This suggests that these compounds may contribute significantly to the antioxidant action of *S. nigrum*.

Overall, the comprehensive investigation of the phytochemical composition, antioxidant activity, and molecular docking analysis enhances our understanding of the therapeutic potential of *S. nigrum* in combating oxidative stress-related diseases such as arthritis, asthma, dementia, and aging. The findings of this study provide a foundation for further research and development of antioxidant-based therapies utilizing natural sources like *S. nigrum* for improved healthcare and well-being.

## Supplementary Material

Supplementary material
