# Supplementary material for: Chemical composition’s effect on Solanum nigrum Linn.’s antioxidant capacity and erythrocyte protection: Bioactive components and molecular docking analysis
Source: Open Life Sci. 2024 Aug 30;19(1):20220944. doi: 10.1515/biol-2022-0944 (PMC11365465; doi:10.1515/biol-2022-0944)
Supplement: Supplementary material [file biol-2022-0944-sm.pdf]

Supplementary material

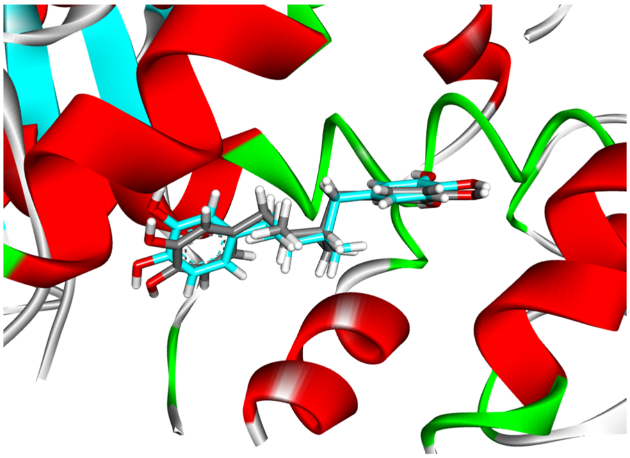

Figure S1: The root means square deviation between the original and docked poses of the cocrystal ligands of Lipoxygenase enzyme (PDB: 6n2w) was 0.62 Å.

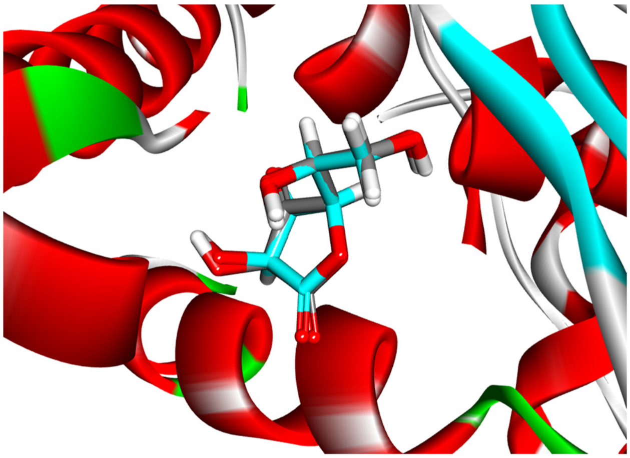

Figure S2: The root means square deviation between the original and docked poses of the cocrystal ligand (Ascorbic acid) of Peroxidase enzyme (PDB: 2x08) was 0.20 Å.

Table S1: Phenolic compounds previously reported and isolated from *Solanum nigrum* L

Quercetin **1** 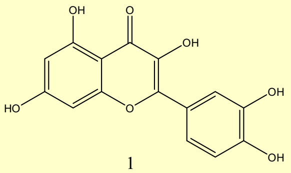 1	Quercitrin **2** 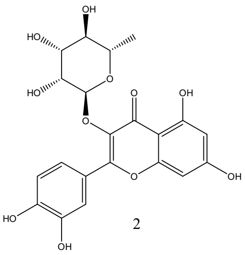 2	Isoquercitrin **3** 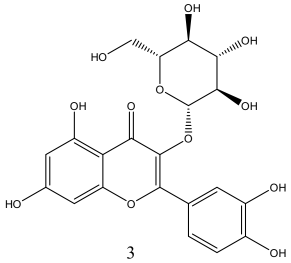 3
Quercetin-3-gentiobioside **4** 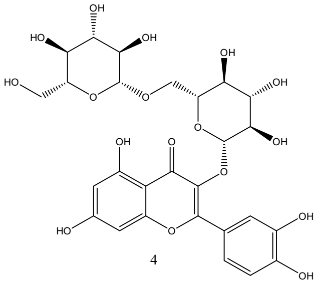 4	6-Hydroxyluteolin 7-sophoroside **5** 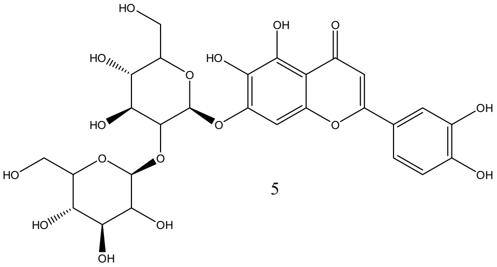 5	
Kaempferol **6** 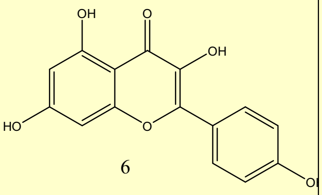 6	Gallic acid **7** 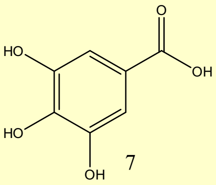 7	2, 4-Dihydroxybenzoic acid **8** 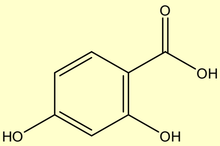 8
Protocatechuic acid **9** 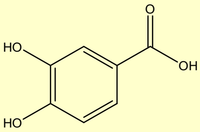 9	Vanillic acid **10** 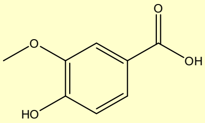 10	4-Hydroxybenzoic acid **11** 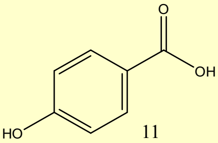 11
2, 5-Dihydroxybenzoic acid (gentisic acid) **12** 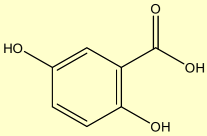 12	Caffeic acid **13** 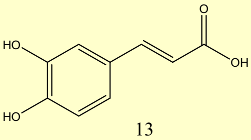 13	Chlorogenic acid **14** 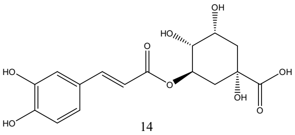 14
4-Hydroxycinnamic acid **15** 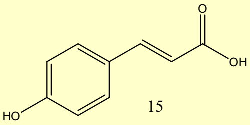 15		

Table S2: Two- and three-dimensional protein/ligand interaction between the ten compounds and arachidonate-5-lipoxygenase (ALOX5, PDB:6n2w)

**Com.No.**	**Lipoxygenase (6n2w)**	
	**2D**	**3D**
Ligand	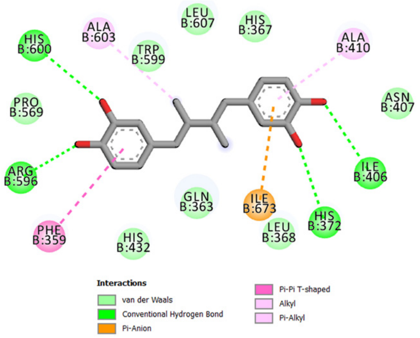	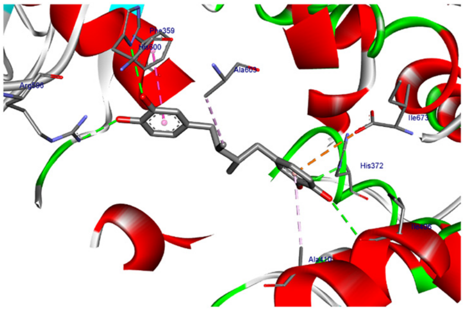
Quercitrin	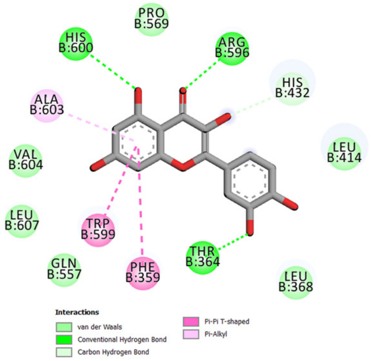	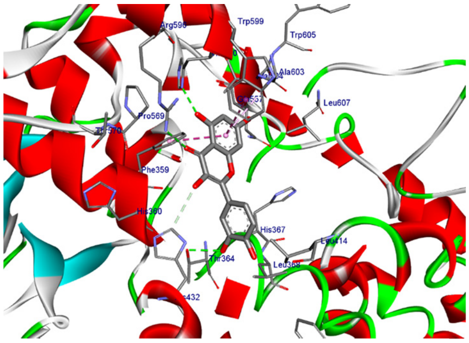
Kaempferol	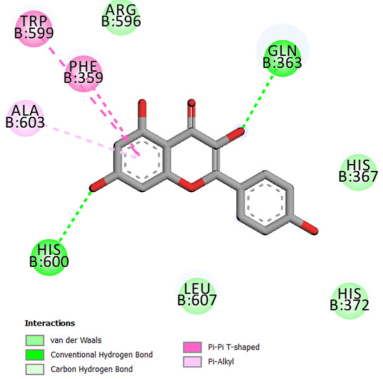	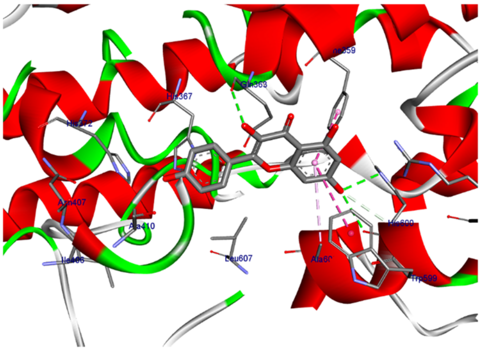
Gallic acid	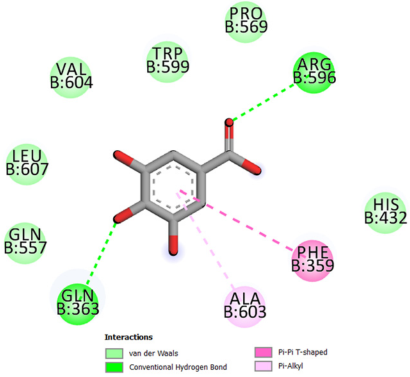	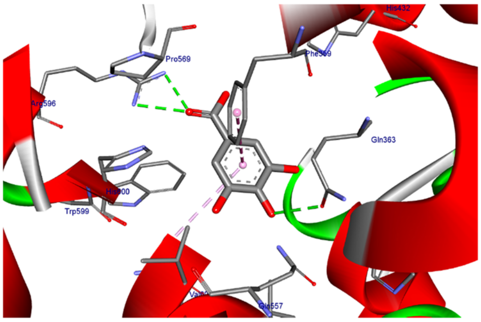

(Continued)

Table S2: *Continued*

Com.No.	Lipoxygenase (6n2w)	
	**2D**	**3D**
2, 4-Dihydroxybenzoic acid	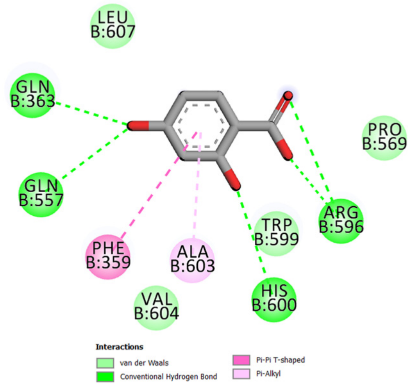	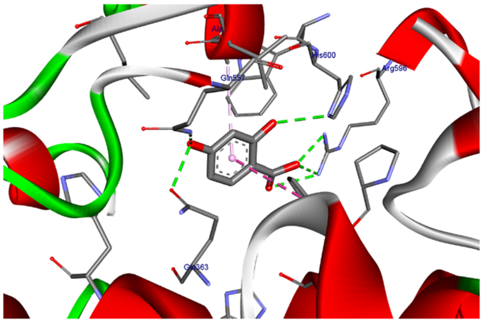
Protocatechuic acid	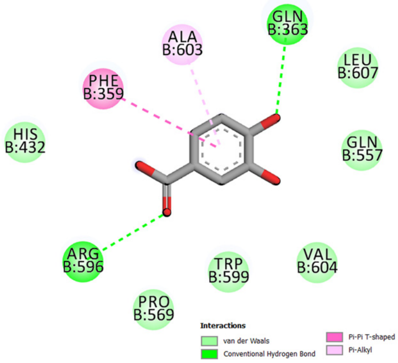	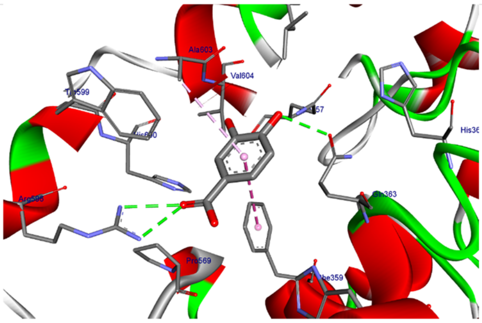
Vanillic acid	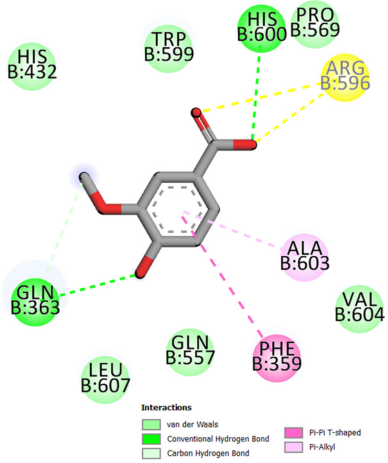	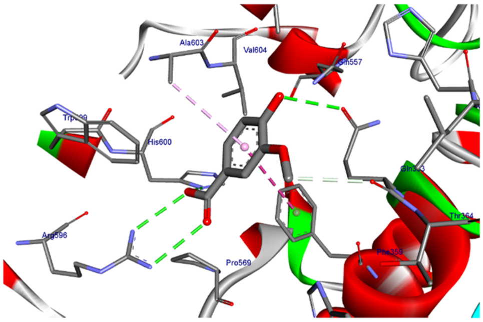
4-Hydroxybenzoic acid	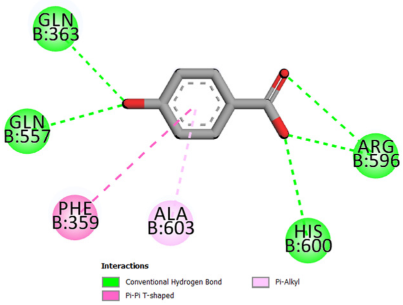	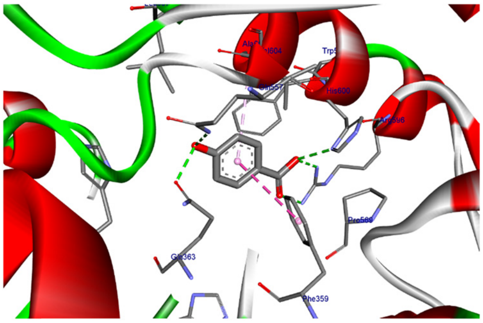

(Continued)

Table S2: *Continued*

Com.No.	Lipoxygenase (6n2w)	
	2D	3D
2, 5-Dihydroxybenzoic acid (gentisic acid)	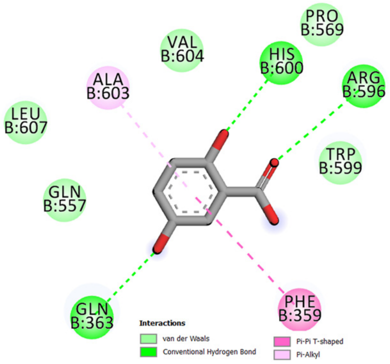	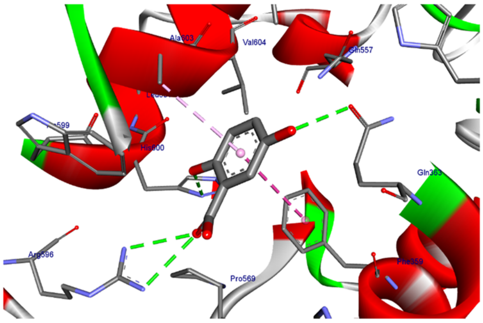
Caffeic acid	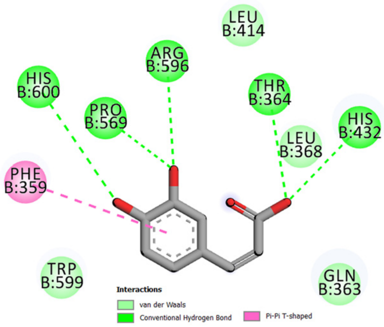	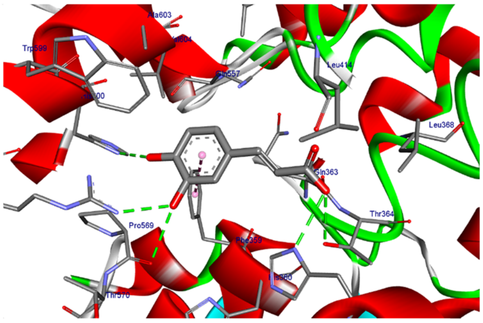
4-Hydroxycinnamic acid	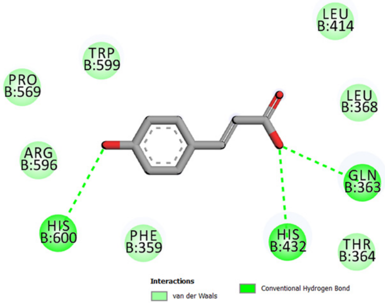	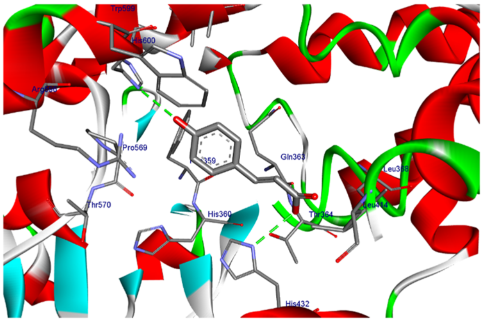

Table S3: Two- and three-dimensional protein/ligand interaction between the ten compounds Cytochrome c Peroxidase (PDB: 2x08)

Co.No.	Peroxidase (2x08)	
	2D	3D
Ascorbic acid	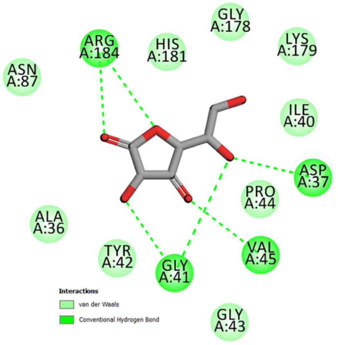	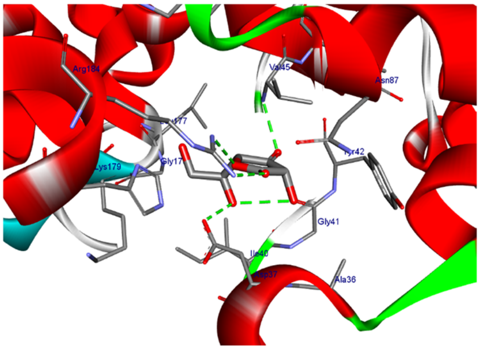
Quercitrin	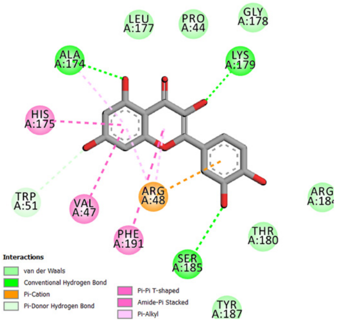	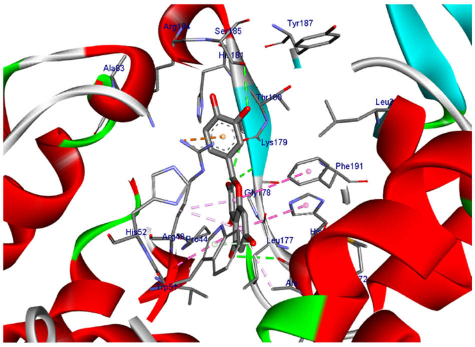
Kaempferol	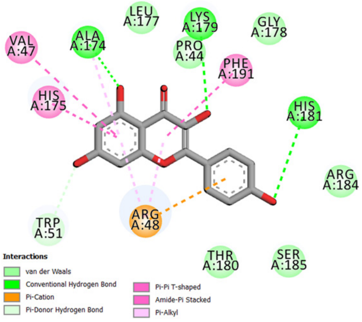	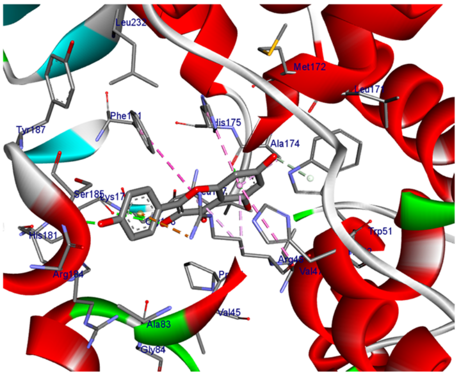
Gallic acid	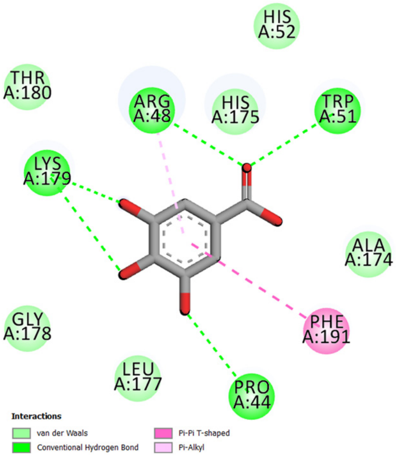	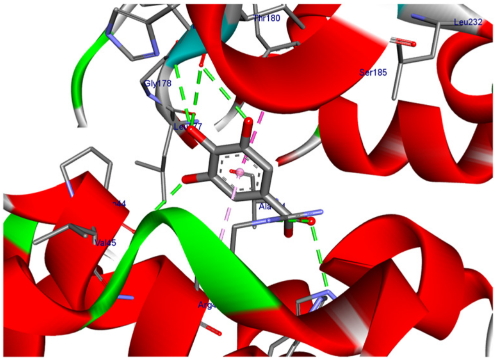

(Continued)

Table S3: *Continued*

<table>
<tr><th rowspan="2">Co.No.</th><th colspan="2">Peroxidase (2x08)</th></tr>
<tr><th>2D</th><th>3D</th></tr>
<tr><td>2, 4-Dihydroxybenzoic acid</td><td>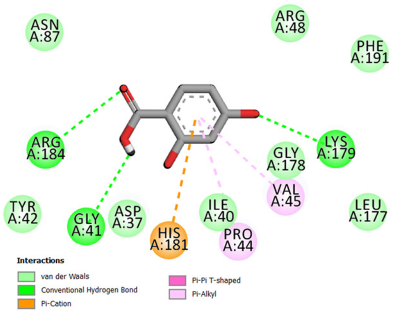
</td><td>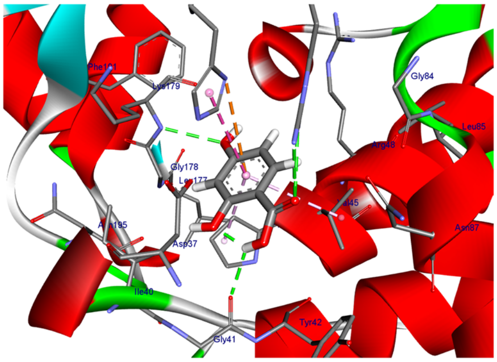</td></tr>
<tr><td>Protocatechuic acid</td><td>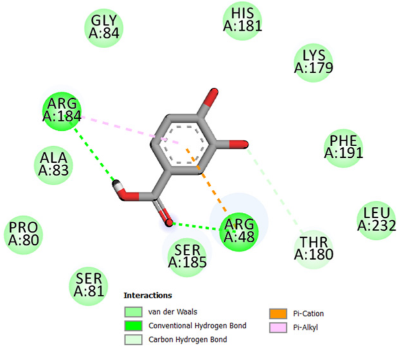
</td><td>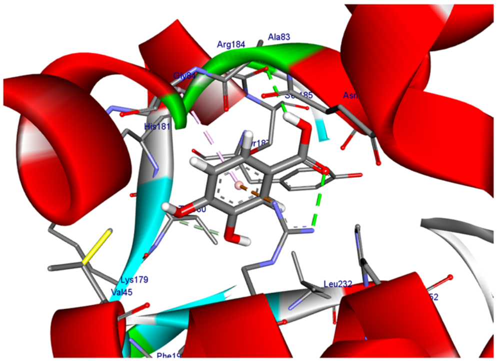</td></tr>
<tr><td>Vanillic acid</td><td>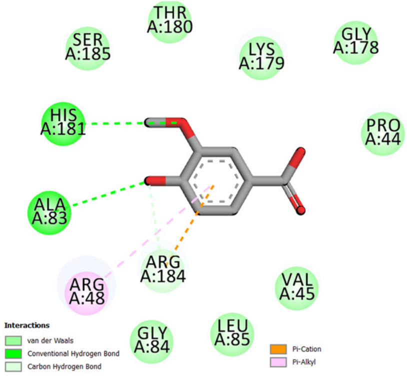
</td><td>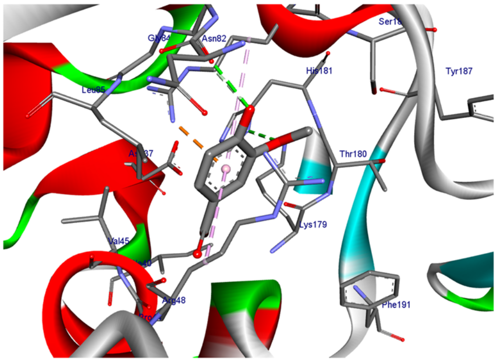</td></tr>
<tr><td>4-Hydroxybenzoic acid</td><td>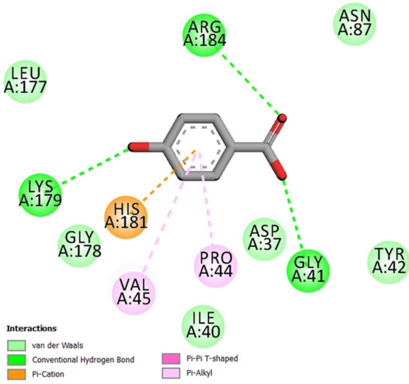
</td><td>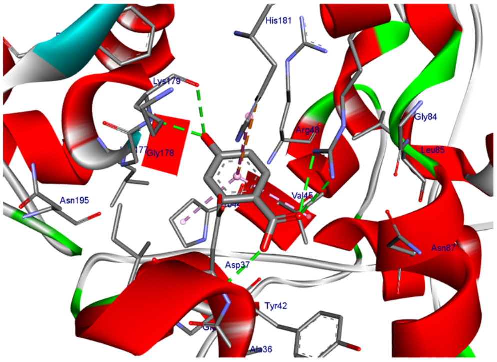</td></tr>
</table>

(Continued)

Table S3: *Continued*

<table>
<tr><th>Co.No.</th><th colspan="2">Peroxidase (2x08)</th></tr>
<tr><th></th><th>2D</th><th>3D</th></tr>
<tr><td>2, 5-Dihydroxybenzoic acid (gentisic acid)</td><td>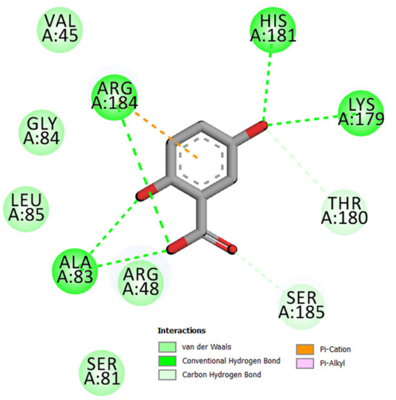
</td><td>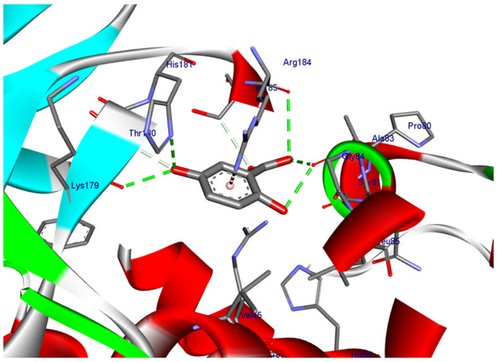</td></tr>
<tr><td>Caffeic acid</td><td>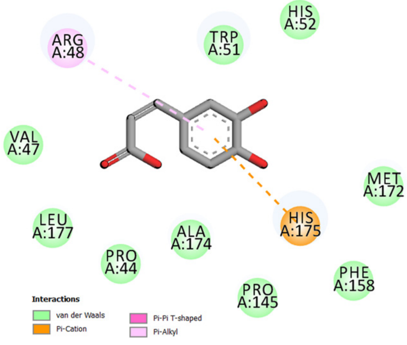
</td><td>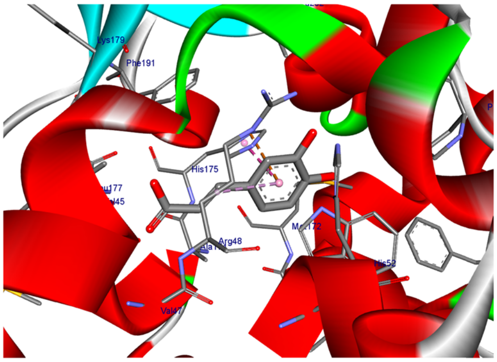</td></tr>
<tr><td>4-Hydroxycinnamic acid</td><td>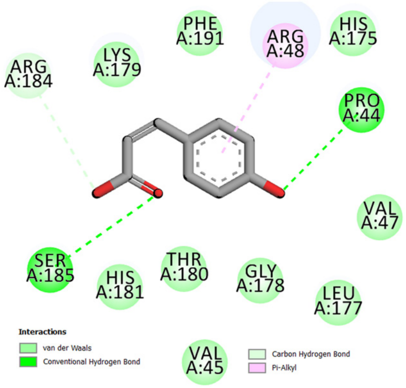
</td><td>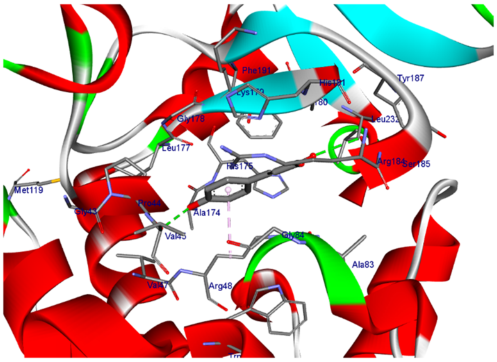</td></tr>
</table>
